# Meta-analysis defines predominant shared microbial responses in various diseases and a specific inflammatory bowel disease signal

**DOI:** 10.1186/s13059-022-02637-7

**Published:** 2022-02-23

**Authors:** Haya Abbas-Egbariya, Yael Haberman, Tzipi Braun, Rotem Hadar, Lee Denson, Ohad Gal-Mor, Amnon Amir

**Affiliations:** 1 Sheba Medical Center, Tel-HaShomer, affiliated with the Tel-Aviv University, Tel Aviv, Israel; 2 Cincinnati Children’s Hospital Medical Center and the University of Cincinnati College of Medicine, Cincinnati, OH USA; 3 The Infectious Diseases Research Laboratory, Sheba Medical Center, Tel-Hashomer, and the Department of Clinical Microbiology and Immunology, Tel Aviv University, Tel Aviv, Israel

**Keywords:** Gut microbiome, Crohn’s disease, Ulcerative colitis, 16S amplicon, Meta-analyses

## Abstract

**Background:**

Gut microbial alteration is implicated in inflammatory bowel disease but is noted in other diseases. Systematic comparison to define similarities and specificities is hampered since most studies focus on a single disease.

**Results:**

We develop a pipeline to compare between disease cohorts starting from the raw V4 16S amplicon sequence variants. Including 12,838 subjects, from 59 disease cohorts, we demonstrate a predominant shared signature across diseases, indicating a common bacterial response to different diseases. We show that classifiers trained on one disease cohort predict relatively well other diseases due to this shared signal, and hence, caution should be taken when using such classifiers in real-world scenarios, where diseases are intermixed. Based on this common signature across a large array of diseases, we develop a universal dysbiosis index that successfully differentiates between cases and controls across various diseases and can be used for prioritizing fecal donors and samples with lower disease probability. Finally, we identify a set of IBD-specific bacteria, which can direct mechanistic studies and design of IBD-specific microbial interventions.

**Conclusions:**

A robust non-specific general response of the gut microbiome is detected in a large array of diseases. Disease classifiers may confuse between different diseases due to this shared microbial response. Our universal dysbiosis index can be used as a tool to prioritize fecal samples and donors. Finally, the IBD-specific taxa may indicate a more direct association to gut inflammation and disease pathogenesis, and those can be further used as biomarkers and as future targets for interventions.

**Supplementary Information:**

The online version contains supplementary material available at 10.1186/s13059-022-02637-7.

## Introduction

Gut microbial imbalance is noted in inflammatory bowel diseases (IBD) [1–3]. However, studies spanning different countries failed to report consistent unified microbial alterations [4], and while altered microbiome likely plays a role in IBD pathogenesis, the precise microbial dysfunction is not entirely understood. In parallel, many other disease-control studies, most of which are not linked with chronic gut inflammation, have shown alteration in the gut microbial composition. Those various microbial shifts across diseases emphasize the lack of systematic understanding regarding the role of the gut microbiota in healthy and disease states, and its link with the overt gut inflammation seen in IBD. Specifically, we still lack a comprehensive understanding whether different diseases like IBD are characterized by distinct microbial shifts that may be therapeutically targeted or rather by a non-specific general “sick-microbial” state [5].

We re-analyzed raw data from publicly available V4 16S amplicon sequences using a unified pipeline (28 diseases, 59 disease cohorts, and 12,838 subjects). We defined 731 bacterial amplicon sequence variants (ASVs) that were associated with a disease state in at least one disease cohort, and by examining the behavior of these ASVs across all disease cohorts, we identified a robust nonspecific microbial response shared by many diseases. Random forest disease-classifiers trained on one disease cohort (i.e., IBD) also predicted relatively well the disease state in other cohorts with different diseases, likely due to this shared microbial pattern, and we used it to define a novel universal dysbiosis index (UniDI) that successfully differentiates between most cases and controls across diseases. Finally, we identified a set of UC/CD-specific taxa that show a pronounced change in UC/CD compared to other diseases. Those can be prioritized for laboratory exploration to study their potential role in eliciting the intestinal inflammation seen in IBD and can direct future IBD-specific interventions.

## Results

### Uniform case-control analytic pipeline

We identified 59 case-control studies that used V4 16S rRNA amplicon sequencing spanning 28 diseases and 12,838 subjects from different geographical regions including North America, Europe, Middle East, and Asia (Table 1, Additional file 1: Table S1). Raw reads from all samples were trimmed and denoised using Deblur. We included two large studies with multiple diseases: the UK Twins and the American Gut Project (AGP) cohorts. In those and other studies where several diseases were investigated, samples were split to specific disease cohorts, with controls randomly divided between the different disease cohorts and using only one sample per patient. Since all cohorts used the same V4 16S rRNA region, we were able to process, combine, and analyze all cohorts together at the ASV sequence level, thus leading to an enhanced phylogenetic resolution compared to taxonomy-based comparisons, which are usually limited to the genus level for 16S amplicon sequencing. Study-specific variation was a major confounder (Additional file 1: Fig. S1A), likely due to sample collection, processing methods [36], and differences in the populations studied. This was further supported by measuring distances (beta-diversity) between sample-groups within and between studies (Additional file 1: Fig. S1B), showing significantly higher distances between studies.

**Table 1:** Description of case control comparison included in our analyses

Disease	Study	Cases	Controls	Country	Reads (mean)	Reads (median)
Anorexia (*n* = 1)	Mack, Cuntz [6]	55	54	Germany	46637	47815
Autism (*n* = 2)	American Gut Project	135	179	USA	16676	14735
	Zurita, Cardenas [7]	27	31	Ecuador	9805	9792
Autoimmune diseases (*n* = 1)	American Gut Project	298	522	USA	15891	14541
Alzheimer (*n* = 1)	Vogt, Kerby [8]	25	25	USA	97726	96469
Bipolar (*n* = 2)	American Gut Project	6	11	USA	15095	14835
	Evans, Bassis [9]	115	64	USA	19952	20540
Cancer (*n* = 2)	UK Twins Project	22	23	UK	74459	60149
	American Gut Project	220	405	USA	15024	13786
*C. difficile* infection (*n* = 1)	American Gut Project	32	61	USA	15718	14641
Chronic fatigue syndrome (*n* = 1)	Giloteaux, Goodrich [10]	49	39	USA	74627	71306
Depression (*n* = 2)	American Gut Project	272	388	USA	15552	14197
	UK Twins Project	75	40	UK	75927	67004
Diabetes T1 (*n* = 1)	Cinek, Kramna [11]	71	103	Multi	15300	13815
Diabetes T2 (*n* = 4)	American Gut Project	36	64	USA	15224	13805
	Kaplan, Wang [12]	349	275	Multi	15804	14155
	UK twins project	20	21	UK	64289	57280
	Li, Chang [13]	19	39	China	45028	46362
Gastroenteritis (*n* = 2)	Braun, Di Segni [14]	203	31	Israel	21453	16920
	Castano-Rodriguez, Underwood [15]	75	423	Australia	33723	29980
Gout (*n* = 1)	UK Twins Project	17	16	UK	64756	54688
Heart diseases (*n* = 2)	American Gut Project	106	168	USA	16411	13559
	UK Twins Project	32	31	UK	74859	65651
Hepatitis B (*n* = 1)	Liu, Li [16]	35	33	China	56086	55684
HIV (*n* = 4)	Cook, Fulcher [17]	41	79	USA	34830	34421
	Dillon, Lee [18]	18	14	USA	71275	74700
	Lozupone, Li [19]	22	12	USA	10506	9562
	Vujkovic-Cvijin, Sortino [20]	82	84	Netherlands	46028	44876
Hypertension (*n* = 1)	UK Twins Project	124	74	UK	69815	61608
IBD (*n* = 2)	American Gut Project	107	194	USA	14866	13099
	UK Twins Project	13	17	UK	64397	59889
IBD-Crohn's Disease (*n* = 7)	American Gut Project	31	58	USA	16298	14187
	Braun, Di Segni [1]	37	29	Israel	22712	19035
	Contijoch, Britton [21]	144	35	multi	61224	59415
	^a^Gevers, Kugathasan [2]	479	116	USA	21719	16229
	Ijaz, Quince [22]	23	70	UK	26140	15395
	Shaw, Bertha [23]	15	10	USA	34801	32161
	Zhou, Xu [24]	183	68	China	4219	4075
IBD-Ulcerative Colitis (*n* = 5)	American Gut Project	39	66	USA	16149	16209
	Contijoch, Britton [21]	109	35	multi	58202	57492
	^a^Gevers, Kugathasan [2]	85	29	USA	22600	21069
	Mar, LaMere [25]	30	13	USA	103111	91547
	Zhou, Xu [24]	73	69	China	3974	3948
Irritable bowel syndrome (*n* = 3)	American Gut Project	444	717	USA	15510	13368
	Pozuelo, Panda [26]	110	66	Spain	37606	37624
	UK Twins Project	21	22	UK	60589	58436
Lupus (*n* = 1)	Luo, Edwards [27]	14	17	USA	27159	25961
Obesity (*n* = 4)	American Gut Project	503	436	USA	15842	13939
	de la Cuesta-Zuluaga, Corrales-Agudelo [28]	172	269	Colombia	30051	25461
	Vangay, Johnson [29]	24	151	multi	4507	3500
	UK Twins Project	33	40	UK	68091	64462
Pancreatitis (*n* = 1)	Zhu, He [30]	145	35	China	18158	17881
Parkinson’s (*n* = 3)	Heintz-Buschart, Pandey [31]	46	37	Germany	14819	14880
	Hill-Burns, Debelius [32]	213	135	USA	9561	9050
	Wallen, Appah [33]	524	316	USA	66616	66459
Psoriasis (*n* = 1)	UK Twins Project	43	42	UK	66034	61304
Rheumatoid arthritis (*n* = 1)	UK Twins Project	34	40	UK	74062	64753
Schizophrenia (*n* = 2)	Nguyen, Kosciolek [34]	18	20	USA	21810	17053
	Xu, Wu [35]	44	40	China	69747	70013
Total 28 disease	Total 59 case-control comparisons	6337	6501			

^a^Studies using biopsies

**Fig. 1:**
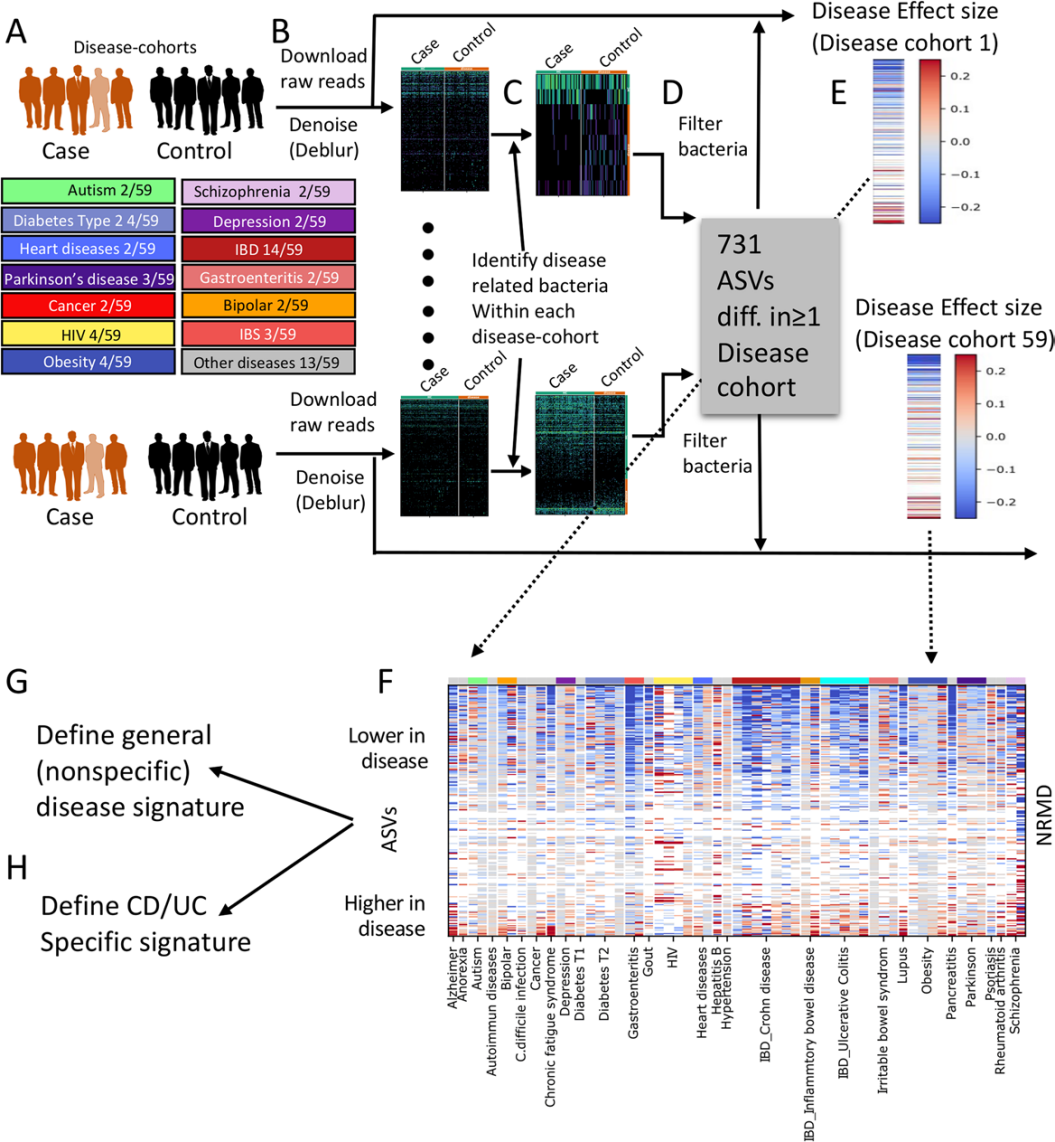
Meta-analysis pipeline. We reanalyzed 12,838 human gut samples, spanning 59 disease cohorts linked with 28 unique diseases (**A**). Per-sample V4 16S amplicon sequencing raw reads were processed using Deblur, resulting in bacterial amplicon sequence variants (ASVs) (**B**). Potential disease dependent ASVs within each original study were identified separately within each disease cohort (rank mean tests, FDR < 0.25, using a random subset of 23 cases and 10 controls to include as many case/control comparison and avoid dominance of large cohort size) (**C**). ASVs were then combined, resulting in 731 unique ASVs (**D**). For each disease cohort, the effect size (normalized rank mean difference—NRMD) was calculated for these 731 ASVs using all available samples in each cohort (*n* = 12,838) (**E**), and results were combined to a single table (**F**). Each column in the heatmap represents a single disease cohort, and each row represents a single ASV, with color representing the NRMD effect size; red and blue colors indicate higher or lower in disease respectively, while white indicates ASVs not present in the disease cohort. Non-disease specific ASVs were identified using a binomial test (FDR < 0.1) (**G**), whereas CD/UC specific ASVs were identified using rank-mean test (FDR < 0.1) (**H**)

To overcome these study-specific signals and facilitate comparison across disease cohorts, we devised a per-study effect size-based pipeline (Fig. 1). The concept is to calculate the case/control effect-size for each ASV within each disease cohort separately, and then perform the meta-analysis on these effect-sizes rather than on the ASV frequencies. Specifically, we first identified ASVs showing a potential case/control difference in at least one study. Since this initial screening is used to identify ASVs that are then further selected using additional statistical tests, and in order to prevent bias in ASV identification due to different cohort sizes, we selected a small subset of samples per study, with a high FDR (dsFDR< 0.25 [37]), in order to include as many studies and differentially abundant ASVs as possible (Fig. 1). Seven hundred thirty-one bacterial ASVs showing significant disease association in at least one cohort were combined (Fig. 1 heatmap), and the effect size [labeled herein as normalized rank mean difference (NRMD)] was now calculated between cases and controls within each disease cohort, using all samples in each disease cohort (*n* = 12,838).

### Similarities and differences in the microbial composition within and between disease states

**Fig. 2:**
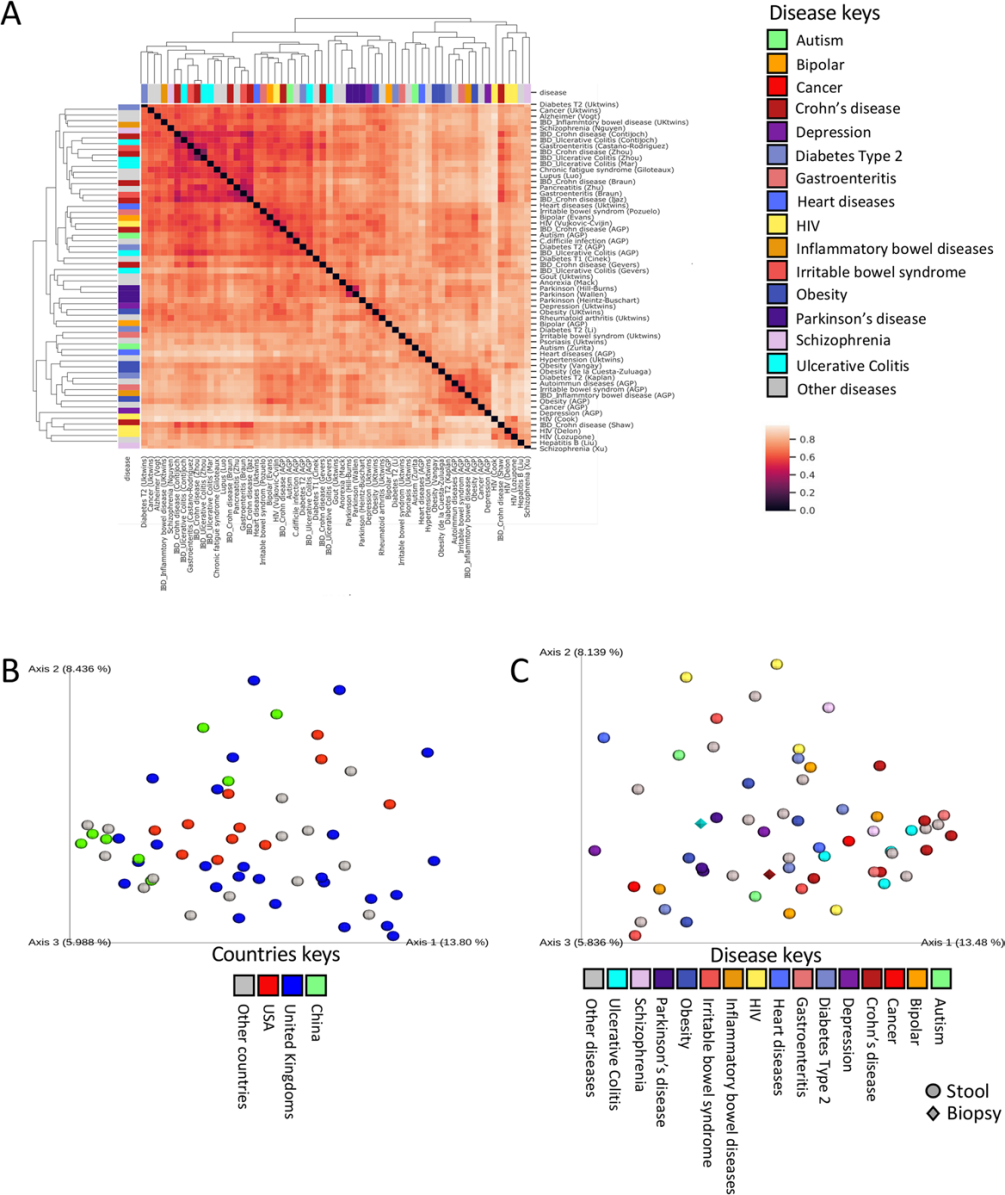
Similarities and differences in the microbial composition between diseases. Modified Bray-Curtis distance matrix was calculated using the 731 ASV effect size ratios between cases and controls across the different disease cohorts using all samples (*n* = 12,838). Comparisons were performed between two disease cohorts only on ASVs found in both. This modified Bray-Curtis metric was used to build the distance matrix (**A**). Darker color indicates high similarity and bright color indicates low similarity. Bar colors on *X* and *Y* axes indicate the specific disease of each disease cohort, as indicated in the disease key. CD UC and IBD are all colored in dark-red but the labeling specifically indicates the disease type; however, those tend to intermix. IBD represent studies in which patients were only labeled as IBD rather than CD or UC. This matrix was then used for the generation of a principal coordinate analysis (PCoA) depicting disease cohort similarity (**B**, **C**), where disease cohorts are colored by country (**B**) or specific disease (**C**)

To evaluate the ability of our pipeline to reduce the study specific contribution and to capture signals across diseases/studies, we used a Bray-Curtis based principal coordinates analysis (PCoA) either on the original mean ASVs relative abundance in cases and controls in each study (Additional file 1: Fig. S1C-D) or on the NRMD effect size resulting from our pipeline. Using the original mean relative abundance, cases, and controls from each disease cohort tended to be positioned together (Additional file 1: Fig. S1C). In contrast, when using the effect size (NRMD) based distances, we were able to eliminate much of the cohort specific signal, with the signal from the different cohorts being spread-out (Additional file 1: Fig. S1D). The full NRMD-based distance matrix (Fig. 2A) shows clustering according to disease rather than study. For example, three Parkinson studies from 3 different cohorts (2 from the US and 1 from Europe) showed high similarities with each other (Fig. 2A). IBD studies were significantly enriched in the left main dendrogram branch (chi-squares *p* value = 0.009, 11/28 vs. 3/30), and CD and UC disease cohorts seemed to intermix. However, 17 other disease cohorts including Alzheimer, lupus, and autism also clustered on the left dendrogram branch. Coloring of the NRMD-based PCoA by country of origin or disease shows that cohorts from the same geographic region are spread through the graph (Fig. 2B) and that different IBD cohorts or different Parkinson cohorts cluster together by disease type (Fig. 2C).

### Non-specific microbial alteration associated with multiple pathologies

**Fig. 3:**
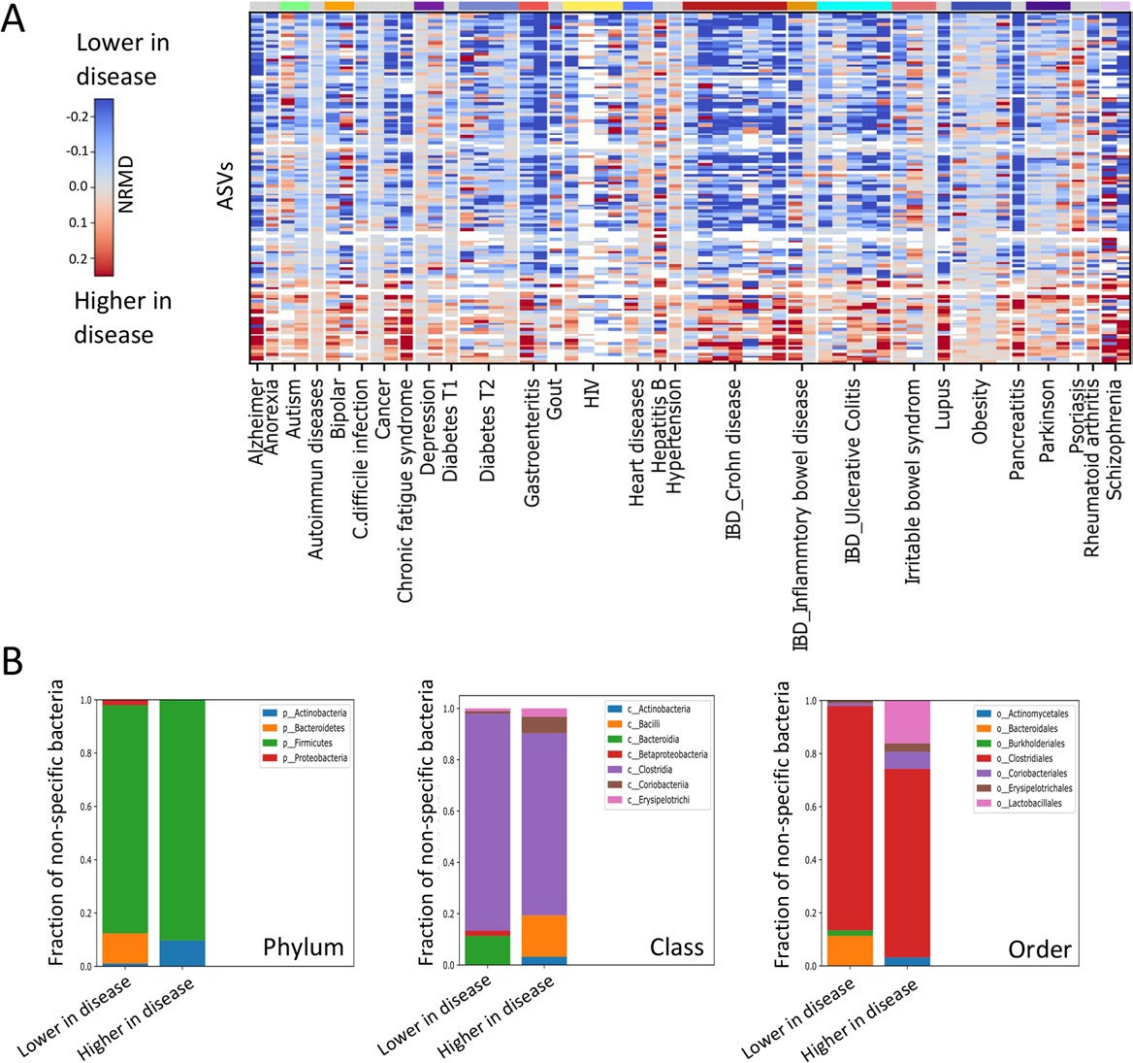
Non-specific microbial signal shared across diseases. **A** Heatmap showing 128 non-specific ASVs identified by applying a binomial test on the ratios of the 731 ASVs across all diseases (FDR < 0.1). Columns are disease cohorts, and rows represent the non-specific ASVs that were significantly changed in at least four different diseases, with colors representing the NRMD. Red and blue indicate higher or lower abundance in disease respectively, while white indicates ASVs not present in the study. **B** Non-specific ASVs were separated to two groups; those lower in disease (76% of non-specific ASVs, left column), and those that are higher in disease (24% of non-specific ASVs, right column). The fraction of bacteria from each of these groups is shown for three taxonomic levels as indicated: phylum, order, and class

To define those non-specific general changes, we searched for ASVs whose NRMD effect size direction significantly differed from a 0.5/0.5 binomial distribution. This resulted in 128 ASVs that have similar behavior across multiple diseases (two sided binomial test with dsFDR< 0.1, Fig. 3A) and therefore associated with general disease state. Most (97 ASVs) showed reduced abundance, while only 31 showed higher abundance across different diseases (Fig. 3A and Additional file 2: Dataset S1). Specifically, we use the direction of the effect (rather than the effect size) for the binomial test used to identify the non-specific bacteria and therefore give equal weight to all studies not depending on the amplification level of the bacteria within each study. The relative taxonomy composition of the two groups showed that *Bacteroidetes* comprise 11 of the 97 nonspecific health-associated ASVs, whereas none were within the 31 disease-associated ASVs (chi-square *p* = 0.01, Fig. 3B). In contrast, *Actinobacteria* taxa (chi-square *p* = 0.02) were more abundant within disease associated ASVs (3/31) compared to health-associated ASVs (1/97) (Fig. 3B). *Lactobacillales* order (phylum: *Firmicutes*) were also significantly enriched within the disease-associated ASVs (5/31 vs. 0/97 for disease- and health-associated ASVs respectively, chi-square *p* < 0.001). To validate the use of the NRMD effect size calculation approach, we reanalyzed the same studies using two additional approaches: (i) rarifying each sample across all studies to 4000 reads/sample (Additional file 1: Fig. S2A, Additional file 2: dataset S1) and (ii) using the LEFSE [38] LDA score instead of the NRMD (Additional file 1: Fig. S2B and Additional file 2: Dataset S1). Those present similar results compared to the NRMD approach, with the mean effect size (i.e., higher, or lower in disease) showing similar direction for 31/31 of the disease-associated and 96/97 health-associated bacteria using the rarified data and 30/31 of the disease-associated and 95/97 health-associated bacteria using the LEFSE LDA score (see supplementary methods for details).

To infer microbial functions enriched in the health vs. disease-associated ASV bacteria, we applied Picrust2 [39] and identified 10 KEGG functions that were more common in disease, and 14 more common in controls (rank-mean test with dsFDR < 0.1, Additional file 1: Fig. S3 and Additional file 3: Dataset S2). Functions enriched in disease associated ASVs included carbohydrate metabolism, whereas functions enriched in health associated ASVs included metabolism of cofactors and vitamins, and amino acid metabolism. Interestingly, cellular community pathways  and more specifically quorum sensing genes, were more common in disease associated ASVs process allowing bacterial populations to communicate and coordinate group behavior, a process which is more commonly used by pathogens [40].

### IBD-specific microbial alteration is predominantly linked to increased abundance of individual taxa

**Fig. 4:**
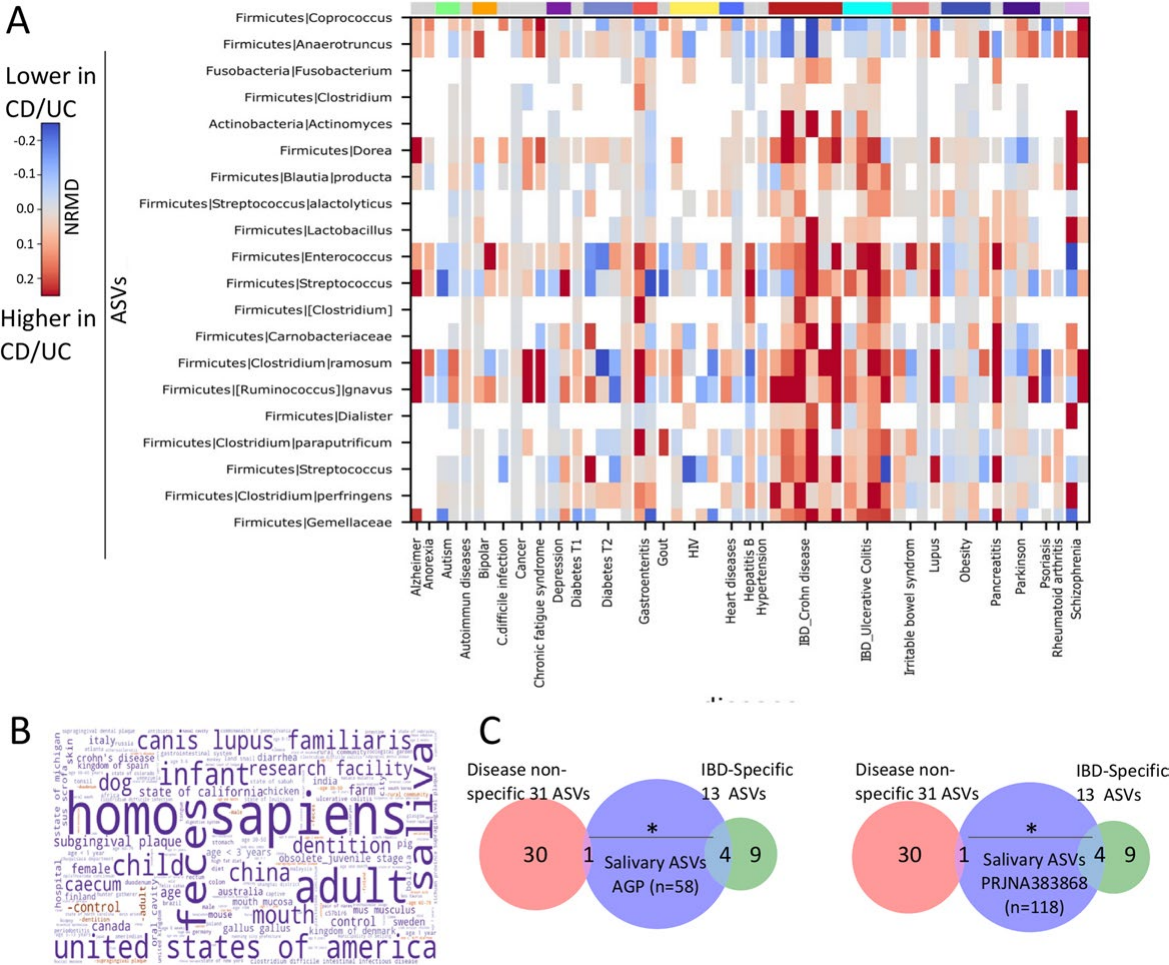
Salivary bacteria are enriched in samples from Ulcerative colitis and Crohn’s disease. **A** Heat map showing 15 CD/UC “specific” ASVs with significantly higher (or lower) effect size in fecal samples of CD and UC cases compared to controls in comparison to other disease cohorts [rank-mean test on the NRMD effect sizes in 10 fecal CD and UC studies compared to other disease (*n* = 45)]. Columns are disease cohorts, and rows represent the CD/UC specific ASVs with colors representing the NRMD; red indicates higher abundance and blue indicates lower abundance in cases vs. controls, and white indicates ASVs not present in the study. **B** A word cloud was generated using dbBact (http://dbbact.org/) [41] using the increased UC/CD-specific bacteria, indicating that UC/CD-specific increased bacteria has been previously found in fecal and saliva human samples. **C** Venn diagram showing overlap between the 31 increased non-specific ASVs and 13 IBD-specific ASVs (red and green circles respectively) salivary obtained samples including those ASVs that are present 25% and above of the samples (blue) identified from other cohorts [AGP (left) and PRJNA38386 [42] (right) see methods section for additional details], emphasizing significant larger overlap between the IBD-specific ASVs and salivary ASVs (chi-square *p* < 0.05) in contrast to the disease non-specific increased ASVs

We next searched for ASVs specifically associated with CD/UC. For that, the naïve approach of performing differential abundance on CD/UC samples compared to controls without considering the behavior in other disease cohorts does not suffice. We therefore defined CD/UC-specific ASVs as ASVs showing significantly higher or lower NRMD effect size in fecal samples from CD and UC disease cohorts (*n* = 10) compared to other non-IBD disease cohorts (*n* = 45) (using permutation-based rank mean test of the per-disease cohort NRMDs, with dsFDR = 0.1). Fifteen ASVs were significantly related to UC and CD, with 13 showing a higher NRMD between UC and CD cases and controls and 2 showing a decrease in NRMD between UC/CD and controls (Fig. 4A and Additional file 4: Dataset S3). Those specific CD and UC enriched ASVs included taxa from *Gemellaceae*, *Veillonellaceae*, *Fusobacteriaceae*, and *Streptococcaceae* families. Term enrichments of those 13 CD/UC-associated specific ASVs using dbBact database showed enrichment for microbial taxa seen in saliva samples (Fig. 4B), with more significant overlap with salivary samples in 2 additional studies (Fig. 4C). Attempts to find other disease-specific signals, including for Parkinson’s disease failed, likely due to lack of sufficient number of studies linked with a specific condition.

### Inaccuracy of classifier to differentiate between different disease states

**Fig. 5:**
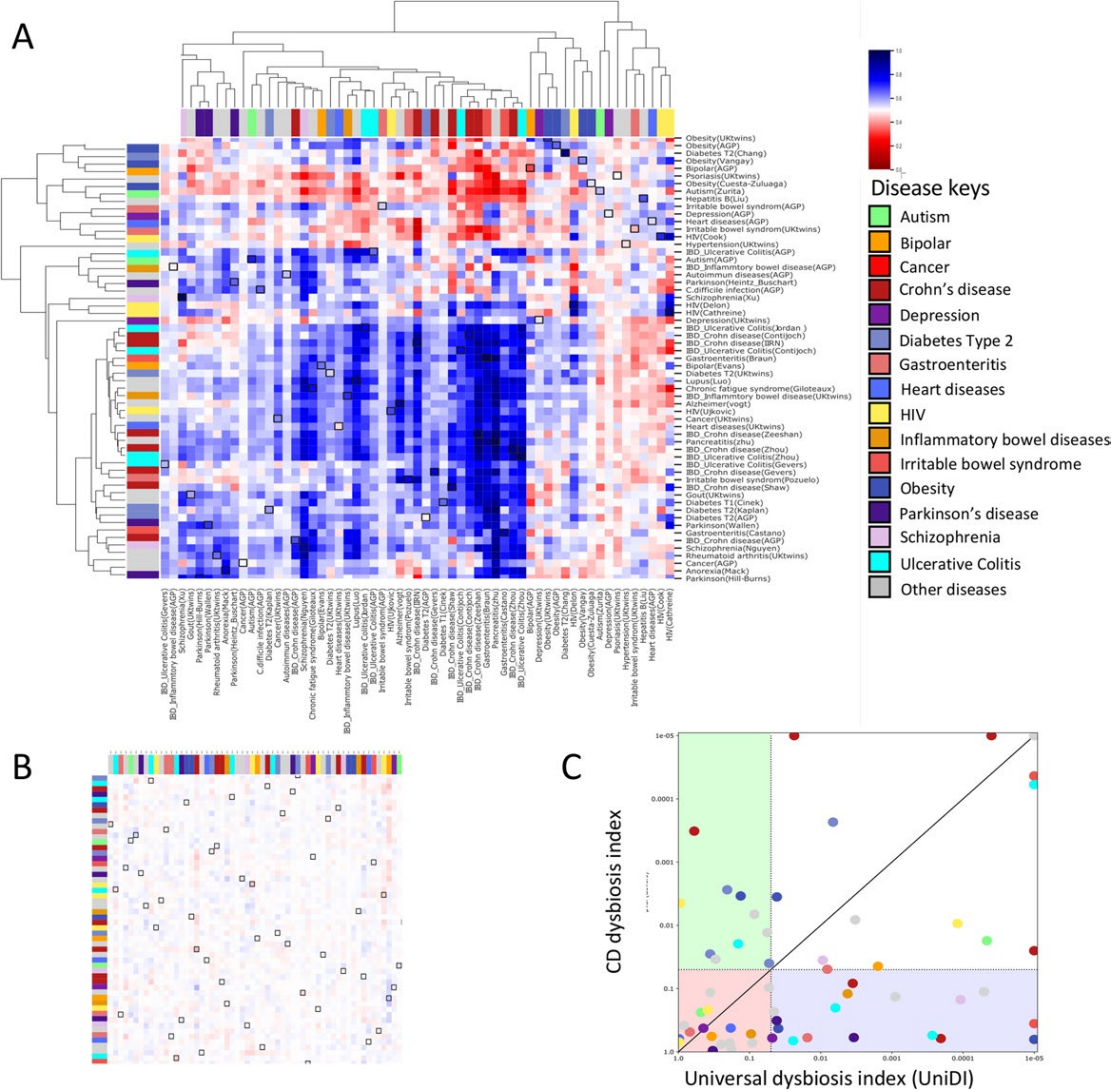
Disease classifier and dysbiosis index predict general microbial signal rather than disease specific signals. **A** Random Forest classifier heatmap, showing the disease/control prediction AUC in each disease cohorts (columns) based on training the classifier on datasets in the different rows (see methods for details). Blue indicates high prediction AUC (> 0.5) and red indicates AUC < 0.5. Training and prediction in each comparison were performed only on shared ASVs between the trained and the predicted cohorts. Squares marked in the heatmap, indicate the prediction results obtained after training of the classifier using the same cohort. **B** Random Forest classifier heatmap, showing the prediction AUC after performing random permutation of labels of the predicting cohort prior to the classifier prediction, to further validate the non-random results obtained in **A**. **C** Dysbiosis index per dataset was measured by two models: per-sample rank (UniDI) [5], and by using the CD dysbiosis index [2], and the resulting *P*-value (Mann-Whitney) after comparing disease and controls for each dataset is shown in the plot where the *x*-axis showing the sample-rank model (UniDI), and the *y*-axis showing the CD dysbiosis index [2]. For the performance evaluation, we used a leave-one-out approach. We iterated over all disease cohorts, and for each iteration, the analysis was performed while leaving out a single validation disease cohort. The *up* _ *nonspecific*, *down* _ *nonspecific* ASV groups were identified as described but without the validation cohort (i.e., the non-specific ASVs were identified based on 58 disease cohorts). Diseases with at least 2 cohorts were colored by a specific color, while single cohorts were all colored in gray (detailed map in Additional file 1: Fig. S5). Pink left down quadrant indicates non-significant *p*-value (p > 0.05) using both UniDI and CD dysbiosis index, green left upper quadrant indicates significant *p*-value (*p* < 0.05) only using CD dysbiosis index, the purple right down quadrant indicates significant *p*-value (*p* < 0.05) only using UniDI, while the larger white quadrant indicates significant *p*-values in both UniDI and CD dysbiosis index. All dot under the diagonal line received lower p value with UniDI while those above had a lower p value with the CD dysbiosis index

Machine learning classifiers are commonly used to differentiate between healthy and disease cases [43]. However, the fact that a large set of bacteria display a consistent change across multiple disease raises the concern that classifiers may capture this shared signal, which may lead to incorrect disease identification and the inability to differentiate between diseases. To test this, we used a supervised learning Random Forests (RF) classifier. For each disease cohort, we separately trained RF to differentiate cases/controls for this disease cohort and then tested its ability to differentiate cases/controls on a different disease cohort. AUC performances are shown in Fig. 5A, where each row represents a disease cohort on which a classifier was trained, and columns represent the disease cohort on which the classifier was tested (when the training and test cohorts were the same, samples were split 2:1 for training/validation). As an estimate for the inherent noise present in the classifier performance, we also tested the performance of the same procedure where the case/control labels of each testing disease cohort were randomly shuffled (Fig. 5B and Additional file 1: Fig. S4). Predicting case/control state in UC and CD by using models built upon other UC and CD cohorts worked relatively well and mostly above what is expected by random. However, models built on other diseases also predicted CD and UC relatively well, and vice-versa, models built on CD and UC were able to predict other diseases. Those results indicate that disease classifiers perform well in identifying sick vs. healthy states but may fail to differentiate between different diseases.

### Universal dysbiosis index (UniDI)

A universal dysbiosis index (UniDI) was built on the identified 128 non-specific ASVs signal. We calculated the per-sample UniDI by rank-transforming the bacteria and computed the normalized log ratio of the sum of the ranks of the 97 health-associated and 31 disease-associated ASVs. We then compared the UniDI (using a leave-one-out approach) to a previously published taxonomy-based CD dysbiosis index [2]. For the performance evaluation, we used a leave-one-out approach: we iterated over all disease cohorts, and for each iteration performed the analysis while leaving out a single validation disease cohort. The *up* _ *nonspecific*, *down* _ *nonspecific* ASV groups were identified as described above (see the “Identification of shared (“non-specific”) ASVs” section), but without the validation cohort (i.e., the non-specific ASVs were identified based on 58 disease cohorts). The resulting UniDI performed better than the CD dysbiosis index, indicating that UniDI can successfully differentiate between most cases and controls across a wide variety of diseases (Fig. 5C, Additional file 1: Fig. S5, and Additional file 1: Fig. S6). Among the UC/CD studies, UniDI showed more significant changes between cases and controls (10/14 studies) than CD dysbiosis index (7/14). Similar results were shown in other diseases such as Parkinson’s; UniDI succeeded to show significant changes in dysbiosis index in 2 out of 3 studies included in the meta-analysis, in comparison to 0 out of 3 in the CD dysbiosis index. These results indicate that UniDI can successfully differentiate between most cases and controls across a wide variety of diseases.

## Discussion

Our meta-analysis used a novel standardized pipeline starting from per-sample raw reads, across diverse pathologic conditions, different parts of the world, 28 diseases, and 12,838 subjects. We focused on studies that used the V4 regions, to facilitate comparison at the ASVs level rather than taxonomy and calculated the per ASV effect size between cases and controls within each original cohort, thus minimizing the study-specific signature. We identified a robust non-specific general disease response dominated by reduction of microbial ASVs (97 ASVs), with a smaller group of 31 bacterial ASVs being over-represented in disease. These non-specific microbial changes may reflect a general response of the body to pathologic conditions and therefore are less likely to be the main source of the chronic gut inflammation seen in IBD. Disease classifiers performed well in identifying many sick vs. healthy states, likely due to this general signal. We define a novel universal dysbiosis index (UniDI) utilizing the shared disease-associated ASVs, that can successfully differentiate between most cases and controls across a wide variety of diseases. Finally, we identified a set of IBD-specific taxa (most of which are salivary bacteria [2], with an increased abundance in IBD), potentially implying a more direct causal association between those and IBD pathogenesis and gut inflammation. Interestingly, recent studies showed that ectopic displacement of oral bacteria in the gut environment resulted in dysbiosis and a decrease in Th17 cells and fecal IgA levels and an increase in the M1/M2 macrophage ratio, thereby promoting chronic inflammation [44].

Meta-analyses systematically compare several independent studies to capture consistent and specific signals across diseases, cohorts, populations, and protocols. The basic unit is the disease cohort rather than samples. Microbiome meta-analysis can take place at several levels; systemic review comparing the results without reanalyzing the raw data [4], reanalyzing the raw data but not focusing on a specific sequence, enabling only comparisons at the taxonomy level [5, 45], and lastly reanalyzing the raw data and focusing on a specific sequence that enables direct comparison within each cohort as performed here. Microbiome-related meta-analyses have already suggested some inconsistencies in bacterial signals in IBD [4, 5, 36]. A thorough systematic review [4] of published results compared 45 articles noted that increase in *Enterobacteriaceae taxa* and a decrease in the butyrate producing *Faecalibacterium prausnitzii* seem to be consistent across studies. Our analyses imply that decrease in *Faecalibacterium prausnitzii* is seen across multiple diseases and are likely not IBD-specific. Another landmark meta-analysis study took the second approach [5], started from the raw data of 28 published case-control studies, that sequenced different regions on the 16S gene (not limited to the V4 region), and also highlighted the depletion of health-associated bacteria across multiple diseases, and some specific microbial signal in colon cancer but not in IBD. We took the third approach, limiting the analyses to similar sequencing region of the 16S (V4). This reduces the number of available studies, but it enables ASV rather than taxonomy-based comparison. For example, 2 different *Clostridium* ASVs showed opposite direction of change (SV14256 increased and SV12376 decreased, Dataset S1) and a similarly opposite pattern was noted for 2 *Ruminococcus* ASVs (SV14506 and SV08112). The non-specific health-associated taxa included *Faecalibacterium prausnitzii* and *Coprococcus* (family: *Lachnospiraceae*) ASVs. In contrast to previous studies, we highlight also ASVs that are induced (rather than only reduced) as part of the non-specific signal including *Clostridium XlVa* (known as *Enterocloster*), *Lactobacillus salivarius*, and *Rothia (family: Micrococcaceae)*. Similarly, a recent meta-analysis of microbiome gene functions of nearly 2000 publicly available fecal metagenomic samples of 7 different diseases also identified many functions that were linked to and overlapped with multiple diseases [46].

Disease-specific microbial ASVs may suggest a more direct link to pathogenesis, and those can be used as biomarkers for diagnostics and for design of potential future interventions. We identified 13 UC/CD-specific ASVs bacteria that were more increased in IBD compared to other diseases. Those included taxa from *Gemellaceae*, *Dialister (*family: *Veillonellaceae), Blautia producta* (family: *Lachnospiraceae*), and *Streptococcus* (family: *Streptococcaceae*). *Gemellaceae* taxa were previously linked with the risk for future CD flare [1]. Additionally, in cystic fibrosis, *Gemella* was also increased during exacerbation and was found to be the most discriminative genus between baseline and exacerbation samples [47]. Similarly, presence of *Streptococcus* in stool samples before CD surgery is predictive of future CD recurrence based on endoscopic scores 6 months after surgery [48]. *Streptococcus* and *Veillonella* can interact metabolically, co-occur in ecosystems, and co-aggregate in biofilms [49], and their combined incubation results in higher IL8 cytokines secretions from dendritic cells [50]. Two of the UC/CD specific ASVs (*Clostridium perfringens* and *Ruminococcus gnavus*) were also part of non-specific signals but those showed significant increase in CD and UC. Those taxa were shown to be specifically reduced during antibiotic treatment in pouchitis IBD patients who responded to treatment, further indicating potential causal associations between those taxa and gut inflammation [51]. Interestingly, many of those IBD-specific bacteria were shown to be present in saliva samples. This may be due to the lower bacterial biomass observed in IBD feces [2, 52, 53], thus leading to an increased fraction of bacteria present in swallowed saliva, or that some of these oral bacteria affect the gut immune response. Further studies are required to clarify these questions.

Disease classifiers are frequently used to identify patients with a given disease. However, since most focus on a single disease and a group of healthy controls, the presence of a strong disease non-specific bacterial response may influence the performance if used outside of the original study in diverse population with different diseases. Our results show that a classifier trained on IBD disease cohort classified with relatively good performance disease/control states in lupus, schizophrenia, or Parkinson’s. This raises the concern that such classifier can incorrectly classify patients with other unrelated disease as cases of the disease of interest. Notably, our results do not preclude the possibility of classifiers to differentiate between different diseases when trained on datasets containing multiple diseases, but rather that it is recommended to train those classifiers also on a large set of diseases.

The concept of a dysbiosis index for estimation of the general host dysbiosis or healthy state was suggested in the landmark IBD study [2] and in recent shotgun sequencing dataset [54]. The recent shotgun sequencing dataset used 4347 human stool metagenomes from 34 published studies across healthy and 12 different disease conditions highlighting 50 bacterial species more prevalent in pooling data from healthy vs. nonhealthy state cases. Once generated, this index can be applied on other shotgun datasets. We expanded this idea using 12,838 subjects, 59 disease cohorts, and 28 diseases, and we generated an index that can be applied to dataset generated using 16S sequencing. We note that an advantage of shotgun compared to 16S sequencing is that shotgun dataset can also inform regarding metabolic pathways and functions, which can be an interesting future approach for to define healthy vs. nonhealthy state pathways across diseases. Our UniDI is shown to perform better than the CD dysbiosis index [2] indicating that UniDI can successfully differentiate between most cases and controls across a wide variety of diseases. One possible use for UniDI is its application as an additional tool for prioritizing fecal microbiota transplant (FMT) donors, where it is desired to obtain samples from donors with lower disease probability. Another application as already suggested [54] for such index is for providing insight into one’s health status from a gut microbiome and for inferring the likelihood of disease independent of the clinical diagnosis. In addition, this index can be calculated per samples and can be included in gut microbial personal report, which can also be followed longitudinally for variations linked with diet and environmental modification in clinical studies.

Our work has several strengths, we included 12,838 subjects, 59 disease cohorts, and 28 diseases from around the world, and the results can be generalizable to those regions. By analyzing only studies covering the V4 region, combined with a unified denoising approach, we combined the different studies at the ASV rather than taxonomic assignments, which are typically limited to the genus level for amplicon sequencing. Additionally, by calculating the within-study disease effect, and using these effect sizes rather than the per-sample abundances, we were able to mitigate biases originating from the different cohorts and experimental methods. Limitations include the need to have controls in each study to capture the effect size, the inability to capture variations between controls, and that it is unlikely but possible that the controls in one cohort have the disease in another cohort. Nevertheless, the grouping of IBD and Parkinson’s disease cohorts from several cohorts using the effect size pipeline is reassuring, and in some of the larger cohort, controls were randomly divided between disease cohorts. Other limitations include the limited metadata characteristics across cohorts and the use of 16S rRNA amplicon sequencing (rather than shotgun data) which limits the ability to identify taxonomy up to the strain levels and the bacteria associated metabolic pathways and functions. All studies selected were based on the same region (V4), which limits the number of available studies. We included a single sample per subject and no longitudinal data, and there may be variations in microbial population overtime that are not captured [52]. However, we and others have shown persistent dysbiosis also in CD and UC patients in remission [1, 55]. While we were able to capture IBD-specific bacteria, using the same approach to identify other disease-specific signals failed, likely due to lack of sufficient studies linked with other diseases. Future analysis will require more disease-specific studies and increased data sharing, as well as additional large population-based studies like UK Twins and the American Gut Project (AGP).

## Conclusions

Despite numerous studies linking the microbiome to human health and disease conditions, there are many gaps regarding which and how those bacteria contribute to specific human disorders. In this meta-analysis, we identified a robust non-specific general response dominated by reduction of microbial ASVs with a smaller group of bacteria that were up-regulated in a large array of diseases. Those non-specific taxa can define a novel universal dysbiosis index (UniDI) that can successfully differentiate between most cases and controls across a wide variety of diseases. Finally, we identified mainly increased IBD-specific taxa, potentially indicating a more direct causal association to pathogenesis and gut inflammation, that can be used as biomarkers and potential future targets for interventions.

## Methods

### Study search and disease cohort selection strategy

We searched for case–control (disease cohort) 16S amplicon sequencing studies using specific keywords in Google Scholar and dbBact (http://dbbact.org/) and by following references in meta-analyses and related case–control studies. Only studies with at least 20 subjects, with stool or biopsies samples that were sequenced using hypervariable V4 region, and for which the per-sample raw FASTA files were publicly available for download or obtained after a specific request, were included. Those studies and sources are summarized in Table 1 and Supplementary Table 1. Only one sample per patient was kept in cases where several samples were obtained. In studies that had UC/CD patients, we considered each disease as a separate disease cohort and randomly divided the controls between the two case-control comparisons. In this meta-analysis, we included two large cohorts with multiple diseases: the UK Twins cohort (https://twinsuk.ac.uk/) and the American Gut Project (AGP) cohort (http://humanfoodproject.com/americangut/). In cases where controls were not defined (such as in the AGP and UK twins), we considered controls as those having BMI < 30 and not taking oral medications other than supplements and vitamins. Controls were then randomly divided (taking into account age, gender, and country of origin when applicable) between the different disease cohorts in each study, so that each control sample participated only in one disease cohort. To define obesity, subjects with BMI 30 or greater were included in the obesity group. We included only subjects with a single disease category and dropped those with two or more diseases, except for IBD patients who were also diagnosed with IBS or autoimmune disease as those may co-exist (and therefore were included in the analysis as IBD patients).

### V4 16S raw data processing

Single-end reads were left trimmed to begin at the end of the 515′F primer and right trimmed to a total length of 150 bp. Reads from each cohort were then aligned and denoised using the Deblur pipeline [56, 57] in qiime2 (qiime2-2019.2 [58]) using default parameters, resulting in a per-study bacterial amplicon sequence variants (ASVs) table. Based on a previous study [59], the AGP included 10 blooming bacteria due to sending samples via postal delivery. Those bacteria were filtered across all studies included in this analysis for consistency in order to prevent sample-storage associated bacteria, and identification of the IBD-specific and non-specific ASV was performed before and after this filtering with very consistent results for the filtered and non-filtered dataset. ASV taxonomic classification was performed using a naive Bayes fitted classifier [60], trained on the August 2013 99% identity Greengenes database, for 150 bp long reads and the corresponding primers set as implemented in qiime2 command: qiime2 feature-classifier classify-sklearn with default parameters.

### Standard (frequency-based) analysis

#### Sample preprocessing

To mitigate the effect of different cohort sizes in different studies, 23 samples were randomly chosen from each case/control group in each disease cohort. In cases where less than 23 samples were available for the group, we used all available samples instead. This resulted in a total of 2356 samples for downstream analysis. In addition, for the aggregated analysis, for each case/control group in each disease cohort all samples (up to 23) were combined into a single aggregate sample by taking the mean frequency for each ASV.

#### PERMANOVA

Samples were rarified to a constant depth of 3000 reads per sample, and sample-sample distances were calculated using the Bray-Curtis and unweighted-unifrac metrics using qiime2. To quantify the contribution of different factors to the microbial composition, PERMANOVA was applied using the Adonis function in the R package Vegan (vegan: Community Ecology Package. R package version 2.5-6.https://CRAN.R-project.org/package=vegan) [61] using both metrics. Variables tested were as follows: case/control, specific disease, country, cohort, disease cohort, and age group (adult/child). Analysis was performed on the original samples as well as on the aggregated samples (i.e., one sample per case/control group in each disease cohort). The total variance explained by each variable was calculated independently of other variables (that is, as the sole variable in the model).

#### Quantification of cohort vs. case/control distance contribution

Bray-Curtis distances between pairs of aggregated samples were calculated for the following pairs: case and control pairs from the same disease cohort, cases from different disease cohorts, and controls from different disease cohorts. The distribution of the distances of these three groups was compared using a two-sided non-parametric Mann-Whitney test.

### Effect size [normalized rank-mean difference (NRMD)] based analysis

#### Selection of ASVs and calculation of the per-ASV effect size

To create the per disease cohort ASV case/control effect table, we used only ASVs that show significant differential abundance (between cases and controls) within at least one disease cohort. These ASVs were identified independently within each disease cohort using a non-parametric rank mean test as implemented in Calour [62] with dsFDR multiple hypothesis correction [37] (FDR < 0.25), based on a random subset of 23 cases and 10 controls samples per disease cohort, in order to avoid the dominance of disease cohorts with a large number of samples (since a larger number of samples can provide higher statistical power). A unified list of ASVs showing potential differential abundance in at least one study was then generated. For each of those ASVs, we then calculated the direction of change and the effect size [normalized rank mean difference NRMD) between the mean of cases and controls] in each case-control comparison using all samples in the disease cohort across all studies (to provide a better estimation of the real effect size). The per-ASV normalized rank-mean difference (NRMD) is scaled to be in the range of − 1 to 1 (independent of the number of samples in each group) and was calculated using the formula:

$$NRMD\left(i,x\right)=\left( mean\left({G}_{case}\left(i,x\right)\right)- mean\left({G}_{control}\left(i,x\right)\right)\right)/\left(\frac{n\left({G}_{case}\left(i,x\right)\right)+n\left({G}_{control}\left(i,x\right)\right)\ }{2}\right)$$

where *NRMD*(*i*, *x*) is the normalized effect size for ASV *x* in disease cohort *i*, *G*_*case*_(*i*, *x*), *G*_*control*_(*i*, *x*) represent the ranked frequencies of the case/control (respectively) for ASV *x* in disease cohort *i*, and *n*(*G*) represents the number of samples in group *G*.

#### NRMD-based beta diversity analysis

To quantify the similarity/dissimilarity between the different disease cohorts, we used the normalized difference between cases and controls in each disease cohort (NRMD). Since not all ASVs are present in all disease cohorts, for each pair of disease cohorts, we calculated a modified Bray-Curtis distance using only the ASVs present in both disease cohorts. The distance was calculated as follows:

$$D\left(i,j\right)=\sum_{\begin{array}{c}x\\ {}x\ is\ in\ disease\ cohort\ i\\ {} and\\ {}x\ is\ in\ disease\ cohort\ j\end{array}}\frac{\mid NRMD\left(i,x\right)- NRMD\left(j,x\right)\mid }{\left| NRMD\left(i,x\right)\right|+\mid NRMD\left(j,x\right)\mid }$$

where *i* and *j* denote two disease cohorts, and *D*(*i*, *j*) is the modified Bray-Curtis distance between these two disease cohorts.

This modified Bray-Curtis distance is similar to the classic Bray-Curtis distance, but calculated only on ASVs present in both samples. We opted for this modified Bray-Curtis metric to reduce the effect of the different disease cohorts (i.e., different populations/extraction protocols etc.) that can lead to the lack of observations of some ASVs in specific cohorts, therefore prohibiting the determination of the NRMD for the ASV in the specific cohort.

A distance matrix for all disease cohort pairs was calculated using this modified Bray-Curtis metric. This matrix was then used for the generation of a PCoA depicting disease cohort similarity using qiime2.

#### Identification of shared (“non-specific”) ASVs

Non-specific ASVs are expected to share the same behavior (i.e., higher in cases or lower in cases) across multiple diseases, whereas for ASVs not associated with a non-specific disease response (the null hypothesis), the direction of change (higher in cases or lower in cases) is expected to follow a 0.5/0.5 binomial distribution. We therefore tested the direction of the effect size (i.e., higher (positive NRMD) or lower (negative NRMD) in cases compared to controls for the given disease cohort) and identified for ASVs whose effect size direction significantly differed from 0.5/0.5 binomial (i.e., ASVs that are higher (or lower) in cases compared to controls in a significant number of different diseases). This was implemented by using a two-sided binomial test (*p* = 0.5) on the sign of the NRMD in the different disease cohorts (only on disease cohorts where the ASV was present), followed by Benjamini-Hochberg FDR control (FDR < 0.1). To prevent bias introduced by diseases represented in multiple disease cohorts, all disease cohorts with the same disease were aggregated to a single entry (prior to the binomial test) with the NRMD defined as the mean of the NRMD of all cohorts with the same disease. The analysis was performed only on ASVs present in at least 4 disease cohorts.

NRMD results validation using additional metrics: NRMD is calculated separately for each disease cohort, and since it is rank based, it should be relatively robust to the number of reads per sample. In order to validate the results obtained using NRMD, we additionally tested for non-specific bacteria using two additional metrics: rarified NRMD and LEFSE-based LDA [38]. Following these additional effect size calculations, we identified the set of non-specific bacteria as described previously (see specific implementation details below). Since significance depends on the *p*-value, which is highly variable even between replicates [63], we also compared the results of LEFSE and rarified NRMD to the basic (non-rarified) NRMD by testing how the direction of change overlap between the two methods on the bacteria identified as non-specific following initial NRMD analysis as follows: for each bacteria identified as significantly non-specific (i.e., higher/lower in multiple disease cohorts compared to healthy controls), we calculated the mean of the LEFSE LDA or rarified NRMD over all disease cohorts and tested whether this mean effect size is positive (higher in disease) or negative (lower in disease). We then counted the number of bacteria for which the basic NRMD and the rarified NRMD (or LEFSE LDA) agree on the change direction. This indicates whether the same behavior (higher or lower in disease) is replicated using the different metrics used.

#### Calculation of rarified NRMD

Prior to NRMD calculation, all samples from all disease cohorts were rarified to 4000 reads per sample. Samples with < 4000 reads were discarded. The NRMD calculation was then performed as described above.

#### Calculation of LEFSE LDA

LEFSE (1.1.01) was used to calculate the LDA for each disease cohort using the parameters -a 1 -l 0 -w 1 (no filtering based on LDA/Wilcoxon or subgroup values). The resulting per-bacteria LDA were converted to positive/negative based on the direction of the higher group (disease/healthy) and were then used for the direction overlap analysis. For the identification of LEFSE LDA based non-specific bacteria, since LEFSE does not provide FDR correction, we used a *p*-value cutoff of 0.1 (as compared to FDR = 0.25 in the NRMD-based analysis).

#### Identification of IBD-specific ASVs

IBD-specific ASVs were defined as ASVs showing significantly higher (or lower) NRMD values in CD and UC fecal disease cohorts in comparison to all other disease cohorts. These IBD-specific ASVs were identified using a rank-mean test (implemented in Calour) on the NRMD in all studies (i.e., 10 CD and UC disease cohorts, vs. 45 disease cohorts with other diseases, not including the 2 non-specific IBD diagnosis disease cohorts, and the 2 disease cohorts that used biopsies rather than fecal samples), with dsFDR correction (FDR = 0.1).

#### dbBact terms word clouds

ASVs (either specific or non-specific), that were shown to be related to a given disease, were compared to all the annotations in the dbbact database to search for ontology terms related to those ASVs (i.e., diseases, geographical locations, bacterial main niches in the body, and habitant for those bacteria). For each term, the word size is the F-score combining the precision (i.e., how many of the query sequences contain the given term) and the recall (i.e., how many of the dbBact annotations containing the term contain the query sequences). Blue and red word colors indicate terms positively or negatively associated with the query sequences respectively.

#### Identification of salivary ASVs in additional studies

Per-sample FASTA reads files were downloaded from the SRA for two studies that included salivary microbiome samples (AGP and PRJNA383868 [42]) sequenced using the V4 region. Sequences were processed using the same pipeline described for the current dataset, and we summarized those ASVs that were present in 25% and more in the salivary samples (those 2 cohorts included 500 and above participants) and looked for overlapped with disease non-specific increased ASVs (*n* = 31) and IBD-specific increased ASVs (*n* = 13).

### Functional enrichment analysis in non-specific bacteria

Functional analysis was performed using per-ASV prediction of KEGG functions obtained using PICRUSt2 [39]. We then searched for KEGG functions present at significantly higher (or lower) fractions in health-associated non-specific bacteria compared to disease-associated non-specific bacteria (e.g., KEGG functions more common in bacteria that increase (decrease) in multiple diseases compared to the bacteria that decrease (increase) in controls). The test was performed on the normalized per-ASV KEGG function table aggregated at KEGG level 2, using the rank-mean test (implemented in Calour) with dsFDR multiple hypothesis correction (FDR < 0.1), comparing relative abundances of KEGG functions in disease-associated vs. health-associated ASVs.

### Classifier building and performance

For the classification, each disease cohort was randomly subsampled to a maximum of 23 cases and 23 control samples. For each pair of disease cohorts (train, predict), ASVs were filtered, keeping only ASVs present in both cohorts. A random forest classifier (implemented in scikit-learn version 0.23.1, using default parameters, 100 trees per forest) was trained on the case/control samples in the training cohort. The trained classifier was then used to predict the case/control status of the prediction cohort, and false and true positive rates and AUC were calculated using scikit-learn. In the cases where the train and predict disease cohorts were the same (i.e., assessing the classifier predictions on the same disease cohort), the disease cohort samples were randomly split to 2/3 of the samples to be used as the training cohort, and the remaining 1/3 of the samples used as the prediction cohort.

Three random null-models were used: in the random-prediction model, labels of the prediction cohort (i.e., case/control) were randomly permuted prior to the classifier prediction. In the random-training model, labels of the training cohort (again case/control) were randomly permuted prior to the training (thus leaving intact the case/control differences in the prediction cohort). In the third model, labels of the prediction cohort were randomly permuted prior to the classifier prediction, and the labels of the training cohort were randomly permuted prior to the training.

AUC values shown represent the mean AUC results of 50 repeats of the entire process described (for both real data and randomizations).

### Universal dysbiosis index (UniDI)

For a given sample (containing *r*_*i*_ reads for ASV 1…*n*):

$$S=\left({r}_1,{r}_2,\dots, {r}_n\right),$$

per-sample reads were first rank-transformed, following the method described in [5]:

$$\overset{\sim }{S}=\mathit{\operatorname{rank}}(S)$$

and the dysbiosis index is then defined as the normalized log ratio of the sum of the ranks of the up and down regulated ASVs:

$$NSDI(S)={\mathit{\log}}_2\left(\frac{\sum_{i\in up\_ nonspecific}\overset{\sim }{s_i}}{\sum_{i\in down\_ nonspecific}\overset{\sim }{s_i}}\bullet \frac{\mid down\_ nonspecific\mid }{\mid up\_ nonspecific\mid}\right)$$

where *NSDI*(*S*) is the non-specific dysbiosis index of sample *S*, and *up* _ *nonspecific* and *down* _ *nonspecific* represent the group of disease nonspecific ASVs higher and lower in cases vs. controls respectively, as described in the paper.

The taxonomy based dysbiosis index was calculated following the method described in [2], implemented for the denoised data using the taxonomy assigned to each ASV:

$$DTAX(S)={\mathit{\log}}_2\left(\frac{\sum_{i\in up\_ taxonomies}{s}_i}{\sum_{i\in down\_ taxonomies}{s}_i}\right)$$

where *up* _ *taxonomies*, *down* _ *taxonomies* are the lists of taxonomies identified as higher and lower in Crohn’s disease, as listed in [2].

For the performance evaluation, we used a leave-one-out approach. We iterated over all disease cohorts and for each iteration performed the analysis while leaving out a single validation disease cohort. The *up* _ *nonspecific*, *down* _ *nonspecific* ASV groups were identified as described above (see the “Identification of shared (“non-specific”) ASVs” section) but without the validation cohort (i.e., the non-specific ASVs were identified based on 58 disease cohorts). The dysbiosis index was then calculated for all samples of the validation cohort, and the *p*-value (for the null hypothesis of similar dysbiosis index distribution for cases and controls in the validation cohort) was tested using the non-parametric Mann-Whitney test with the single sided hypothesis (cases > controls).

### Data and code availability

Accession numbers for all studies used in the analysis are available in Additional file 1: supplementary table S1.

Commands and Scripts used for the generation of the per-study biom table and code are available in the sites Github and zenodo [41, 64].

## Supplementary Information

**Additional file 1: Supplementary Table.** Accession numbers for the studies used in the meta-analyses. **Figure S1.** The pipeline used for microbial characterization in our study overcomes cohort specific determinants and enables comparisons between disease cohorts. **Figure S2.** Non-specific microbial signal shared across diseases after rarefaction, and after using Lefse LDA. **Figure S3.** Prediction of the non-specific KEGG ontologies microbial signal shared across diseases. **Figure S4.** Disease classifier fails to predict control and disease samples when shuffling the samples before prediction. **Figure S5.** Dysbiosis index using UniDI and CD dysbiosis index. **Figure S6.** Dysbiosis index generated by the per-sample rank method (UniDI) showed more significant values between case/control samples.

**Additional file 2: Dataset S1.** Non-specific ASVs signal (separate excel file) using initial NRMD, rarified NRMD (to 4K reads/sample), and LEFSE LDA.

**Additional file 3: Dataset S2.** Per-ASV prediction of KEGG functions (PICRUSt2) significantly higher (or lower) in disease-associated non-specific bacteria.

**Additional file 4: Dataset S3.** IBD-specific ASVs.

**Additional file 5.** Review history.

## Data Availability

Studies’ accession numbers are available in Additional file 1: table S1. Scripts used for the generation of the per-study biom table, and code are available in the sites Github and zenodo [41, 64].
