# Supplementary material for: Meta-analysis defines predominant shared microbial responses in various diseases and a specific inflammatory bowel disease signal
Source: Genome Biol. 2022 Feb 23;23:61. doi: 10.1186/s13059-022-02637-7 (PMC8867743; doi:10.1186/s13059-022-02637-7)
Supplement: Supplementary file 1 — Additional file 1: Supplementary Table. Accession numbers for the studies used in the meta-analyses. Figure S1. The pipeline used for microbial characterization in our study overcomes cohort specific determinants and enables comparisons between disease cohorts. Figure S2. Non-specific microbial signal shared across diseases after rarefaction, and after using Lefse LDA. Figure S3. Prediction of the non-specific KEGG ontologies microbial signal shared across diseases. Figure S4. Disease classifier fails to predict control and disease samples when shuffling the samples before prediction. Figure S5. Dysbiosis index using UniDI and CD dysbiosis index. Figure S6. Dysbiosis index generated by the per-sample rank method (UniDI) showed more significant values between case/control samples. [file 13059_2022_2637_MOESM1_ESM.docx]

**Meta-analysis identifies shared microbial responses in various diseases and specific inflammatory bowel disease signal**

Short title: Meta-analysis of the gut microbiome

Haya Abbas-Egbariya^1^^, Yael Haberman^1,2^^*, Tzipi Braun^1^, Rotem Hadar^1^, Lee Denson^2^, Ohad Gal-Mor^3^, Amnon Amir^1^*

^First equal contribution

^1^ Sheba Medical Center, Tel-HaShomer, affiliated with the Tel-Aviv University, Israel.

Tel. (+972)3-530288.

^2^ Cincinnati Children’s Hospital Medical Center and the University of Cincinnati College of Medicine, Cincinnati, OH, USA

^3^The Infectious Diseases Research Laboratory, Sheba Medical Center, Tel-Hashomer, and the Department of Clinical Microbiology and Immunology, Tel Aviv University, Tel Aviv, Israel

*****Corresponding Authors:

Yael Haberman, MD, PhD ([Yael.Haberman@sheba.health.gov.il](mailto:Yael.Haberman@sheba.health.gov.il) & yael.haberman@cchmc.org)

Amnon Amir, PhD ([amnonim@gmail.com](mailto:amnonim@gmail.com)).

Sheba Medical Center, Tel Hashomer, Israel

Telephone number: 972-3-5302692

**Additional file 1**

**Supplementary Table**. Accession numbers for the studies used in the metanalyses

**Figure S1.** The pipeline used for microbial characterization in our study overcomes cohort specific determinants and enables comparisons between disease-cohorts.

**Figure S2.** Non-specific microbial signal shared across diseases after rarefaction, and after using Lefse LDA.

**Figure S3.** Prediction of the non-specific KEGG ontologies microbial signal shared across diseases.

**Figure S4.** Disease classifier fails to predict control and disease samples when shuffling the samples before prediction.

**Figure S5.** Dysbiosis index using UniDI and CD dysbiosis index.

**Figure S6.** Dysbiosis index generated by the per-sample rank method (UniDI) showed more significant values between case/control samples.

**Supplementary Table**

**Table S1: Accession numbers for the studies used in the metanalyses**

| **Disease (n=28)** | **Study (n=59)** | **Database used** |
| --- | --- | --- |
| Anorexia (n=1) | Mack, Cuntz [14] | NCBI SRA: PRJEB11199 |
| Autism (n=2) | American Gut Project | Qiita: study ID 10317 |
|  | Zurita, Cardenas [15] | NCBI SRA: PRJEB27306 |
| Autoimmune diseases (n=1) | American Gut Project | Qiita: study ID 10317 |
| Alzheimer (n=1) | Vogt, Kerby [16] | Private request |
| Bipolar (n=2) | American Gut Project | Qiita: study ID 10317 |
|  | Evans, Bassis [17] | Private request |
| Cancer (n=2) | UK Twins Project | Private request |
|  | American Gut Project | Qiita: study ID 10317 |
| *C. difficile* infection (n=1) | American Gut Project | Qiita: study ID 10317 |
| Chronic fatigue syndrome (n=1) | Giloteaux, Goodrich [18] | NCBI SRA: PRJEB13092 |
| Depression (n=2) | American Gut Project | Qiita: study ID 10317 |
|  | UK Twins Project | Private request |
| Diabetes T1 (n=1) | Cinek, Kramna [19] | NCBI SRA: PRJNA445932 |
| Diabetes T2 (n=4) | American Gut Project | Qiita: study ID 10317 |
|  | Kaplan, Wang [20] | EMBL-EBIENA: ERP117287 |
|  | UK twins project | Private request |
|  | Li, Chang [21] | NCBI SRA: ERP107659 |
| Gastroenteritis (n=2) | Braun, Di Segni [22] | NCBI SRA: PRJEB15447 |
|  | Castano-Rodriguez, Underwood [23] | NCBI SRA: PRJEB23690 |
| Gout (n=1) | UK Twins Project | Private request |
| Heart diseases (n=2) | American Gut Project | Qiita: study ID 10317 |
|  | UK Twins Project | Private request |
| Hepatitis B (n=1) | Liu, Li [24] | GSE108847 |
| HIV (n=4) | Cook, Fulcher [25] | NCBI SRA: PRJNA422134 |
|  | Dillon, Lee [26] | NCBI SRA: PRJNA227062 |
|  | Lozupone, Li [27] | NCBI SRA: ERP003611 |
|  | Vujkovic-Cvijin, Sortino [28] | NCBI SRA: PRJNA589036 |
| Hypertension (n=1) | UK Twins Project | Private request |
| IBD (n=2) | American Gut Project | Qiita: study ID 10317 |
|  | UK Twins Project | Private request |
| IBD-Crohn's Disease (n=7) | American Gut Project | Qiita: study ID 10317 |
|  | Braun, Di Segni [29] | NCBI SRA: PRJNA450540 |
|  | Contijoch, Britton [30] | NCBI SRA: PRJNA413199 |
|  | *Gevers, Kugathasan [13] | NCBI SRA: PRJNA205152 and PRJNA237362 |
|  | Ijaz, Quince [31] | NCBI SRA: PRJEB18780 |
|  | Shaw, Bertha [32] | NCBI SRA: SRP076281 |
|  | Zhou, Xu [33] | NCBI SRA: PRJEB22028 |
| IBD-Ulcerative Colitis (n=5) | American Gut Project | Qiita: study ID 10317 |
|  | Contijoch, Britton [30] | NCBI SRA: PRJNA413199 |
|  | *Gevers, Kugathasan [13] | NCBI SRA: PRJNA205152 and PRJNA237362 |
|  | Mar, LaMere [34] | NCBI SRA: PRJNA313074 |
|  | Zhou, Xu [33] | NCBI SRA: PRJEB22028 |
| Irritable bowel syndrome (n=3) | American Gut Project | Qiita: study ID 10317 |
|  | Pozuelo, Panda [35] | NCBI SRA: PRJNA268708 |
|  | UK Twins Project | Private request |
| Lupus (n=1) | Luo, Edwards [36] | NCBI SRA: PRJNA352037 |
| Obesity (n=4) | American Gut Project | Qiita: study ID 10317 |
|  | de la Cuesta-Zuluaga, Corrales-Agudelo [37] | NCBI SRA: PRJNA417579 |
|  | Vangay, Johnson [38] | NCBI SRA: PRJEB28687 |
|  | UK Twins Project | Private request |
| Pancreatitis (n=1) | Zhu, He [39] | NCBI SRA: SRP125273 |
| Parkinson’s (n=3) | Heintz-Buschart, Pandey [40] | NCBI SRA: PRJNA381395 |
|  | Hill-Burns, Debelius [41] | NCBI SRA: ERP016332 |
|  | Wallen, Appah [42] | NCBI SRA: prjna601994 |
| Psoriasis (n=1) | UK Twins Project | Private request |
| Rheumatoid arthritis (n=1) | UK Twins Project | Private request |
| Schizophrenia (n=2) | Nguyen, Kosciolek [43] | EBI: ERP107975 |
|  | Xu, Wu [44] | CNGB: CNP0000401 |

**Supplementary Figures**


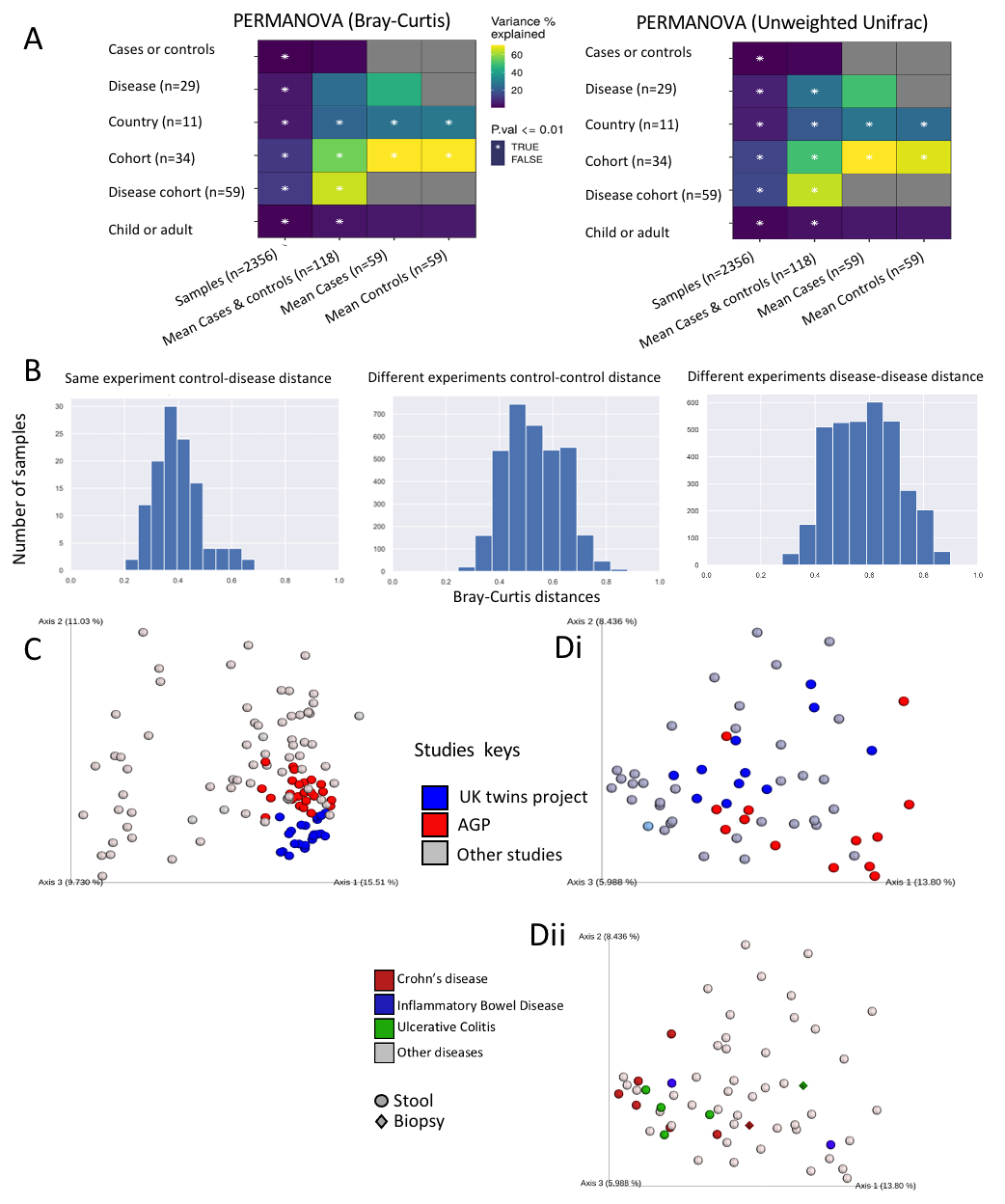


**Figure S1: The pipeline used for microbial characterization in our study overcomes cohort specific determinants and enables comparisons between disease-cohorts.** We reanalyzed 12,838 human gut samples, spanning 59 disease-cohorts comparisons linked with 28 unique diseases. V4 16S amplicon sequencing raw reads were processed separately for each original study using Deblur, resulting in bacterial amplicon sequence variants (ASVs). To evaluate our process before and after applying the effect size within each disease-cohort comparison, we used a subset of up to randomly chosen 23 controls and 23 cases from each disease-cohort to avoid sample size bias, resulting in 2356 samples. **A.** Bray Curtis PERMANOVA (left) and Unweighted Unifrac PERMANOVA (right) were applied to quantify the contribution of different factors affecting the gut microbial composition before we applied the effect size within each disease-cohort comparison. We included all 2356 samples’ ASVs (leftmost column), or only the means obtained in the control and disease groups in each of the 59 disease-cohort (see methods section for details) together for the 59 disease states and controls, and separately for the 59 controls and 59 diseases as indicated. PERMANOVA shows that the original study as well as the specific disease-cohort explains most (44%) of the variation if we do not consider the effect size within each disease-cohort comparison. (* = P ≤ 0.01). Total n is shown in brackets. **B.** Histograms for Bray-Curtis distances between case and control pairs from the same cohort (left), control sample pairs from different cohorts (middle), and case sample pairs from different cohorts (right) indicating higher similarities between disease and controls from the same cohort in comparison to controls from a different cohort, emphasizing the original cohort contribution (Mann-Whitney p-value < 1E-10 for all group comparisons). C-D. PCoA analysis showing Bray Curtis distances for the mean relative abundance of ASVs in each disease-cohort group, before (**C**) and after applying the effect size within each disease-cohort comparison (**Di-ii**), colored by the original studies or disease-cohorts included in the analysis.

**
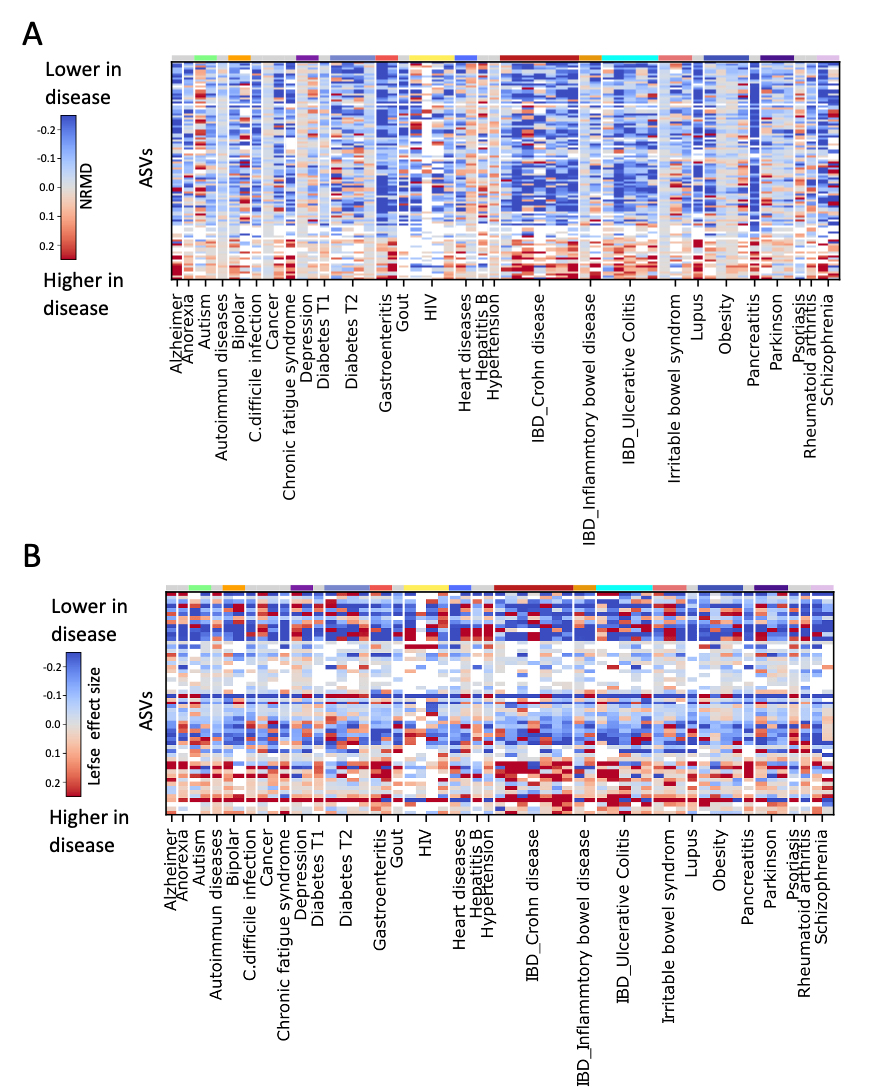
**

**Figure S2. Non-specific microbial signal shared across diseases after rarefaction, and after using Lefse LDA. A.** Heatmap showing 98 (79 lower and 19 higher in disease) non-specific ASVs identified by applying a binomial test on the ratios across all diseases, after rarifying to 4000 reads/sample and using the NRMD metric. **B.** Heatmap showing 55 (41 lower and 14 higher in disease) non-specific ASVs identified by applying a binomial test on the per-study Lefse LDA metric (between healthy and sick). **A-B.** Columns are disease-cohorts, and rows represent the non-specific ASVs that were significantly changed in at least four different diseases, with colors representing the NRMD. Red and blue indicate higher or lower abundance in disease respectively, while white indicates ASVs not present in the study. As an additional validation, we compared to the mean effect size (i.e., higher, or lower in disease) of the original 128 non- specific NRMD signal (97 lower and 31 higher ASVs in disease) and showed similar direction for 31/31 of the disease-associated and 96/97 health-associated bacteria using the rarified data (see supplementary methods for details), and 30/31 of the disease-associated and 95/97 health-associated bacteria using the LEFSE LDA score (see supplementary methods for details).

**
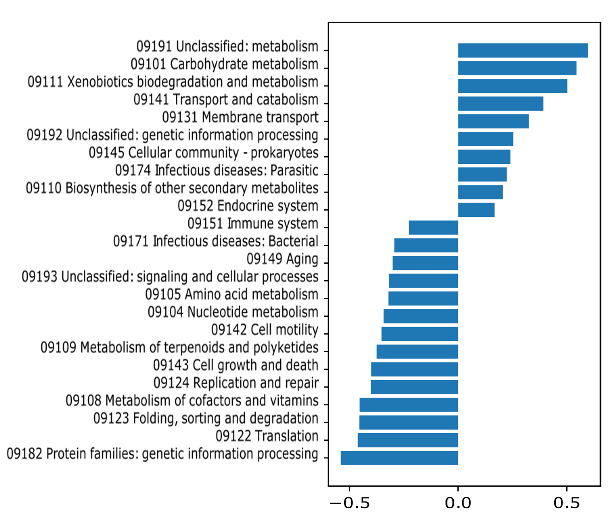
**

**Figure S3: Prediction of the non-specific KEGG ontologies microbial signal shared across diseases.** Functional analysis was performed using per-ASV prediction of KEGG ontologies using PICRUSt2, to infer microbial community functions significantly enriched in non-specific ASVs showing an increase (top) or decrease (bottom) across different diseases.

**
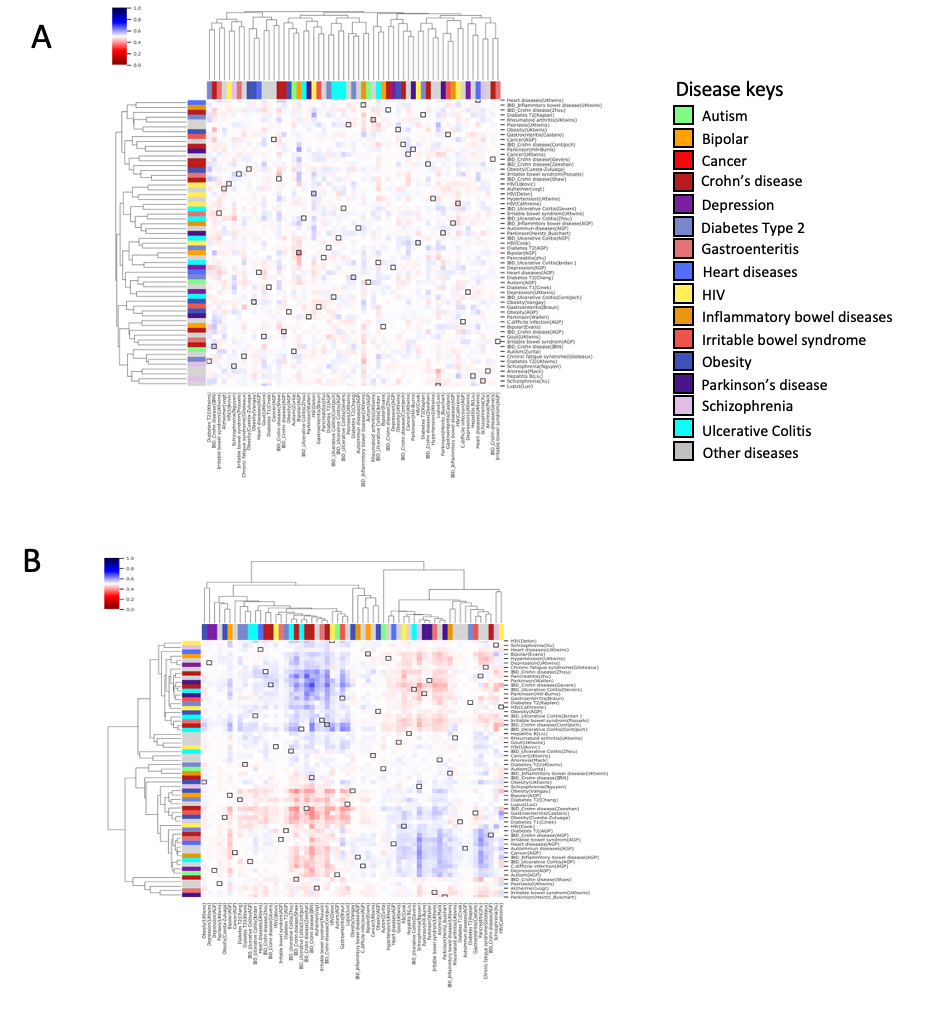
**

**Figure S4: Disease classifier fails to predict control and disease samples when shuffling the samples before prediction.** **A.** Random Forest classifier heatmap, showing the prediction accuracy after performing random permutation of labels of the predicting cohort prior to the classifier prediction, to further validate the non-random results obtained in figure 5A. **B.** Random Forest classifier heatmap, showing the prediction accuracy after performing random permutation of labels of the training cohort prior to the classifier prediction, to further validate the non-random results obtained in figure 5A. Red indicates high prediction AUC and blue indicates AUC < 0.5. Training and prediction in each comparison were performed only on shared ASVs between the trained and the predicted cohorts. Marked squares in the heatmap, indicate the prediction results obtained after training of the classifier using the same cohort.

**Figure S5: Dysbiosis index using UniDI and CD dysbiosis index**. Same as shown in Figure 5C. Each disease- cohort is marked with a reference number. Pink left down quadrant indicates non-significant p-value (p<0.05) using both uniDI and CD dysbiosis index, green left upper quadrant indicates significant p-value only using CD dysbiosis index, the purple right down quadrant indicates significant p-value only using uniDI, while the larger white quadrant indicates significant p-values in both uniDI and CD dysbiosis index.


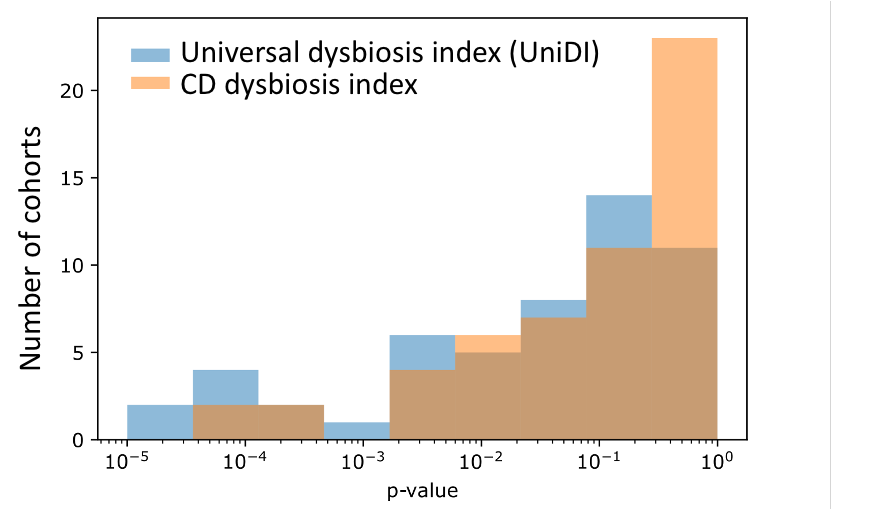


**Figure S6: Dysbiosis index generated by the per-sample rank method (UniDI) showed more significant values between case/control samples:** Dysbiosis index per dataset was measured by two models: per-sample rank [5], and by using CD dysbiosis index [2], and the resulting P-value (Mann-Whitney) for each dataset is shown in the plot where the x-axis showing p-value, and the y-axis showing the number of cohorts.
