# Supplementary material for: Meta-analysis defines predominant shared microbial responses in various diseases and a specific inflammatory bowel disease signal
Source: Genome Biol. 2022 Feb 23;23:61. doi: 10.1186/s13059-022-02637-7 (PMC8867743; doi:10.1186/s13059-022-02637-7)
Supplement: Supplementary file 5 — Additional file 5. Review history. [file 13059_2022_2637_MOESM5_ESM.docx]

Review History

**First round of review**

**Reviewer 1**

**Are you able to assess all statistics in the manuscript, including the appropriateness of statistical tests used?** Yes, and I have assessed the statistics in my report.

**Comments to author:**

General comments

This manuscript follows in the footsteps of Duvallet et al., Nature Communications 2017 and identified general disease-associated bacteria vs. IBD-specific bacteria by using a metanalysis approach. The authors use much more data than the original publications and improved methodology (ASVs not OTUs at genus level, better control of country-study specific effects) and thus (hopefully) end up with more accurate set of disease-specific bacteria for UC and CD (although most of these are still at genus level). They develop a generic dysbiosis index of general disease-associated bacteria (either lower or higher in multiple disease states) using machine learning and then show that it outperforms a CD-specific dysbiosis index. I find the paper to be a useful and important contribution to the microbiome field.

Specific comments

Genus names should be capitalized (see for example page 12). Also in some places the names of family and genus are connected such as "Micrococcaceae Rothia" or "Veillonellaceae Dialister" - it is unclear whether this means the genus within the family and related genera? If not either just state the genus or better yet, write the family in () - Dialister (family: Veillonellaceae )

"characterized by distinct pathobionts shifts that may be therapeutically targeted, or rather by a non-specific general "sick-microbial" state [5]." - this sentence should be revised and the term "pathobiont" - should be avoided as it does more harm than good (see XXYY).

The color palette used for figure 2 makes it nearly impossible to distinguish between Crohn's disease, ulcerative colitis and inflammatory bowel diseases.

What are the "Inflammatory bowel diseases" data-points shown in figure 2? Do they represent studies in which patients were only lableled as "Inflammatory bowel diseases" rather than Crohn's or colitis?

Page 6
"some diseases, such as Parkinson and IBD tended to cluster together", I think that the authors mean that different IBD cohorts or different PD cohorts cluster together (by disease type), and not that PD groups with IBD (which is what their phrasing implies, but is not what one sees in the figure) - maybe edit to "cohorts from some diseases, such as Parkinson or IBD tended to cluster together"

I am not sure the "functional analysis" based on PICRUSt2 has any biomedical meaning, especially since they generalize features from many strains/species and in the end give rather cryptic results. For example, what does "endocrine system" and "aging" mean for bacterial genomes, for example. If the authors must show this, it is best left to supplementary info.

Figure 4A is a little confusing since the colors (hotter meaning higher in disease) make sense but the "lower in disease" and "higher in disease" are too far from the NRMD bar and also opposite to what one expects. I knew what species to expect where, but as it is, it is very confusing.

Page 10

"with a smaller group of 31 bacterial ASVs being up-regulated" should be "with a smaller group of 31 bacterial ASVs being over-represented"
"to pathologic condition" - should be "to pathologic conditions"
Page 11
"have already suggested towards some inconsistencies" - should be "have already suggested some inconsistencies"
"and a similar pposite pattern " should be "and a similarly opposite pattern"
Page 15
"Despite numerus studies" should be "Despite numerous studies"

I find the "Conclusions" section completely redundant, especially since the discussion is quite long
Page 17
"the disease cohort samples were randomly to 2:1" - fix
"and the dysbiosis index is defined" = should be "was defined"
"the nonspecific up and down regulated ASVs" - "change to over and under-represented"
Page 19
some redundancy in the author contribution section
"We gratefully acknowledge Ornit Hall for their suggestions to the manuscript." - fix

Figure 3 title "B. Non-specific ASVs bacteria were separated to two groups" should be "B. Non-specific ASVs were separated to two groups"
Figure 4 title - would have been clearer to day that salivary bacteria are enriched in samples from UC and CD patients
Figure 5: "Disease classifier and dysbiosis index predict general microbial signal rather than disease specific signal." Should be
"Figure 5: Disease classifier and dysbiosis index predict general microbial signal rather than disease specific signals".

**Reviewer 2**

**Are you able to assess all statistics in the manuscript, including the appropriateness of statistical tests used?** Yes, and I have assessed the statistics in my report.

**Comments to author:**

Abbas-Egbariya and colleagues conduct a meta-analysis on multiple 16S rRNA datasets with the aim of defining a novel universal dysbiosis index able to differentiate between cases and controls across different diseases. This is accomplished through a unified pipeline that identifies a nonspecific microbial response shared by multiple diseases. Authors also focuses on identifying taxa specific to discriminate between ulcerative colitis and Crohn's disease patients from healthy subjects.

Although the topic involved in the manuscript is of interest for the microbiome community, I feel that a few methodological details in addition to some biological aspects remain unclear and should be clarified in a revised version of the paper:
1. The datasets included in the analysis are not characterized in terms of their mean sequencing depth, a factor that likely affects the detection of taxa with lower relative abundance. Differences in the sequencing depth could introduce biases in the analysis, especially when cohorts from different studies are analyzed together at the ASV sequence level. I feel that authors should test if results remain valid after rarefaction of the sequencing depth. It is stated in the supplementary material that rarefaction is performed for PERMANOVA analysis, however it is not clear to me if rarefaction is applied also before the effect size calculation.
2. Datasets are also not characterized in terms of processing methods (such as primer types). From my understanding, authors try to overcome such biases by conducting a meta-analysis on the effect-size of each ASV rather than working on the ASV frequencies directly. However, it is still not clear how the meta-analysis on the effect-sizes is really able to control study specific biases, an aspect that should be better stated in the manuscript.
3. Authors define a non-specific microbial alteration associated with multiple pathologies. Due to the use of 16S rRNA data, biological characterization is limited to the identification of taxa that are involved in these non-specific microbial alterations, while it lacks insights on metabolic pathways. Although this issue cannot be solved from this analysis, some discussions on the use of shotgun data should be added into the manuscript.
4. The identification of a universal dysbiosis index (UniDi) that can successfully differentiate between cases and controls (across multiple diseases) is interesting and give insights about a shared microbial response during the pathogenesis process. I feel authors should provide more examples/applications on the use of UniDi besides its application for faecal microbiota transplantation.
5. Effect sizes are calculated through NMRD. What if other strategies are employed for this? I suggest validating findings by considering effect sizes provided by other methods (such as LEfSe: Segata, Nicola, Jacques Izard, Levi Waldron, Dirk Gevers, Larisa Miropolsky, Wendy S. Garrett, and Curtis Huttenhower. "Metagenomic biomarker discovery and explanation." Genome biology 12, no. 6 (2011): 1-18)
6. How does your findings relate to results presented in this paper (Duvallet, Claire, Sean M. Gibbons, Thomas Gurry, Rafael A. Irizarry, and Eric J. Alm. "Meta-analysis of gut microbiome studies identifies disease-specific and shared responses." Nature communications 8, no. 1 (2017): 1-10) that also tried to identify shared microbial responses?
7. Similarly to the previous point, the definition of the UniDi in the current work is quite similar to the definition of the Gut Microbiome Health Index (GMHI) performed in Gupta, Vinod K., Minsuk Kim, Utpal Bakshi, Kevin Y. Cunningham, John M. Davis, Konstantinos N. Lazaridis, Heidi Nelson, Nicholas Chia, and Jaeyun Sung. "A predictive index for health status using species-level gut microbiome profiling." Nature communications 11, no. 1 (2020): 1-16. Please comment on this.

**Authors Response**

**Point-by-point responses to the reviewers’ comments:**

**Point-by-point response to comments from the Editor and Reviewers**

Thank you for the thoughtful review of our manuscript. We have addressed each of the points raised by the reviewers in the revised manuscript, which we believe is significantly improved.

*Reviewer #1: General comments

This manuscript follows in the footsteps of Duvallet et al., Nature Communications 2017 and identified general disease-associated bacteria vs. IBD-specific bacteria by using a metanalysis approach. The authors use much more data than the original publications and improved methodology (ASVs not OTUs at genus level, better control of country-study specific effects) and thus (hopefully) end up with more accurate set of disease-specific bacteria for UC and CD (although most of these are still at genus level). They develop a generic dysbiosis index of general disease-associated bacteria (either lower or higher in multiple disease states) using machine learning and then show that it outperforms a CD-specific dysbiosis index. I find the paper to be a useful and important contribution to the microbiome field.*

**Response:** We thank the reviewer for the review of our manuscript, for highlighting its strength and expansion of the Duvallet et al. paper and for acknowledging its contribution to the microbiome field.

*Specific comments

Genus names should be capitalized (see for example page 12). Also in some places the names of family and genus are connected such as "Micrococcaceae Rothia" or "Veillonellaceae Dialister" - it is unclear whether this means the genus within the family and related genera? If not either just state the genus or better yet, write the family in () - Dialister (family: Veillonellaceae )*

**Response:** We apologize for the inconsistency and corrected those as suggested in the text.

*"characterized by distinct pathobionts shifts that may be therapeutically targeted, or rather by a non-specific general "sick-microbial" state [5]." - this sentence should be revised and the term "pathobiont" - should be avoided as it does more harm than good.*

**Response:** this was modified to **“***characterized by distinct microbial shifts that may be therapeutically targeted, or rather by a non-specific general "sick-microbial" state”*

*The color palette used for figure 2 makes it nearly impossible to distinguish between Crohn's disease, ulcerative colitis and inflammatory bowel diseases.*

**Response:** We thank the reviewer for this comment. The color palette has been modified to better distinguish between Crohn's disease, ulcerative colitis, and inflammatory bowel diseases.

*What are the "Inflammatory bowel diseases" data-points shown  in figure 2? Do they represent studies in which patients were only lableled as "Inflammatory bowel diseases" rather than Crohn's or colitis?*

**Response:** "Inflammatory bowel diseases" represent studies in which patients were only labeled as "Inflammatory bowel diseases" rather than Crohn's or colitis. This has been added to figure 2 legend.

*Page 6
"some diseases, such as Parkinson and IBD tended to cluster together", I think that the authors mean that different IBD cohorts or different PD cohorts cluster together (by disease type), and not that PD groups with IBD (which is what their phrasing implies, but is not what one sees in the figure) - maybe edit to "cohorts from some diseases, such as Parkinson or IBD tended to cluster together"*

**Response:** We thank the reviewer for this comment. This has been modified as suggested.

*I am not sure the "functional analysis" based on PICRUSt2 has any biomedical meaning, especially since they generalize features from many strains/species and in the end give rather cryptic results. For example, what does "endocrine system" and "aging" mean for bacterial genomes, for example. If the authors must show this, it is best left to supplementary info.*

**Response:** We have now moved the PICRUST2 enrichment figure to the supplementary, as suggested (Figure S3). We agree that there are many non-relevant terms using PICRUSt2 but thought that the enrichment of quorum sensing genes in the disease associated ASVs is an interesting observation.

*Figure 4A is a little confusing since the colors (hotter meaning higher in disease) make sense but the "lower in disease" and "higher in disease" are too far from the NRMD bar and also opposite to what one expects. I knew what species to expect where, but as it is, it is very confusing.*

**Response:** Figure 4A has been modified for clarity as suggested.

*Page 10
"with a smaller group of 31 bacterial ASVs being up-regulated" should be "with a smaller group of 31 bacterial ASVs being over-represented"
"to pathologic condition" - should be "to pathologic conditions"
Page 11
"have already suggested towards some inconsistencies" - should be "have already suggested  some inconsistencies"
"and a similar  pposite pattern " should be "and a similarly opposite pattern"
Page 15
"Despite numerus studies" should be "Despite numerous studies"*

*I find the "Conclusions" section completely redundant, especially since the discussion is quite long
Page 17
"the disease cohort samples were randomly to 2:1" - fix
"and the dysbiosis index is defined" = should be "was defined"
"the nonspecific up and down regulated ASVs" - "change to over and under-represented"
Page 19
some redundancy in the author contribution section
"We gratefully acknowledge Ornit Hall for their suggestions to the manuscript." - fix
Figure 3 title "B. Non-specific ASVs bacteria were separated to two groups" should be "B. Non-specific ASVs were separated to two groups"
Figure 4 title - would have been clearer to day that salivary bacteria are enriched in samples from UC and CD patients
Figure 5: "Disease classifier and dysbiosis index predict general microbial signal rather than disease specific signal." Should be
"Figure 5:* Disease classifier and dysbiosis index predict general microbial signal rather than disease specific signals".

**Response:** Those sentences have been all corrected as suggested. We have shortened the conclusions and discussion as recommended by the reviewer, but also added some parts to the discussion as suggested by reviewer2.

*Reviewer #2: Abbas-Egbariya and colleagues conduct a meta-analysis on multiple 16S rRNA datasets with the aim of defining a novel universal dysbiosis index able to differentiate between cases and controls across different diseases. This is accomplished through a unified pipeline that identifies a nonspecific microbial response shared by multiple diseases. Authors also focuses on identifying taxa specific to discriminate between ulcerative colitis and Crohn's disease patients from healthy subjects.

Although the topic involved in the manuscript is of interest for the microbiome community, I feel that a few methodological details in addition to some biological aspects remain unclear and should be clarified in a revised version of the paper:
1. The datasets included in the analysis are not characterized in terms of their mean sequencing depth, a factor that likely affects the detection of taxa with lower relative abundance. Differences in the sequencing depth could introduce biases in the analysis, especially when cohorts from different studies are analyzed together at the ASV sequence level. I feel that authors should test if results remain valid after rarefaction of the sequencing depth. It is stated in the supplementary material that rarefaction is performed for PERMANOVA analysis, however it is not clear to me if rarefaction is applied also before the effect size calculation.*

**Response:** We thank the reviewer for this comment.

We have added the per-study mean and median sequencing depth to Table 1.

The NRMD effect size used in the study is based on the non-rarified data, but the analysis is performed within each cohort, and therefore inter-study difference in read depth are accounted for. Additionally, NRMD uses a non-binary statistic, and hence it is relatively robust to variations in read depth. We therefore opted for using the non-rarified basic-NRMD, as it does not lose power due to discarding reads.

Following the reviewers’ suggestion, we have now also added a validation of the NRMD by using a rarefaction of 4000 reads/sample (Figure S2 and suppl dataset 1). For the non-specific signal we got 98 ASVs (79 lower and 19 higher in disease). Of those lower in disease, 57/79 (72%) overlapped with our initial non-rarified reported ASVs that are lower in disease, and none overlapped with the initial non-rarified 31 ASVs higher in disease (chi square p<0.0001). Similarly, of the ASVs higher in disease, 13/19 (68%) overlapped with the non-rarified reported ASVs higher in disease, and none with the 97 ASVs lower in disease (chi square p<0.0001). Since significance depends on the p-value, which is highly variable even between replicates (see for example Halsey et al 2015), we also compared the results of the rarified-NRMD to the basic-NRMD (non-rarified) by testing the direction-of-change overlap between the two methods (rarified and non-rarified) using the initial 128 bacterial ASVs identified as non-specific following basic-NRMD analysis: for each non-specific bacteria, we calculated the mean of the rarified NRMD over all disease cohorts, and tested whether this mean effect size is positive (i.e. higher in diseases) or negative (i.e. lower in disease). We then counted the number of bacterial ASVs for which the basic-NRMD and the rarified NRMD agree. This indicates whether the same behavior (higher or lower in disease) is replicated using the two different approaches used. Of the 128 bacteria identified as non-specific using the basic-NRMD analysis, 31/31 “higher in disease” and 96/97 “lower in disease” agreed when using the mean rarified NRMD effect size. This has been added to the manuscript. Details of this analysis have been added to the supplementary methods section.

*2. Datasets are also not characterized in terms of processing methods (such as primer types). From my understanding, authors try to overcome such biases by conducting a meta-analysis on the effect-size of each ASV rather than working on the ASV frequencies directly. However, it is still not clear how the meta-analysis on the effect-sizes is really able to control study specific biases, an aspect that should be better stated in the manuscript.*

**Response:** We agree with the limitations pointed out by the reviewer, regarding the different biases arising from per-study processing (such as extraction, PCR conditions, etc.). For example, as shown in Figure S1C, when using a naïve Bray-Curtis metric (i.e., based on bacterial frequencies in each sample), the effect of the different studies is much larger than the disease/control difference. The analysis method we introduce (within-study NRMD, and the following analysis on these NRMDs), is intended to overcome some of the batch effect of the different studies, since we are looking at the difference between disease and healthy samples originating from the same study and thus processed using the same protocol. As shown in figure S1D, following this method, much of the cohort-specific signal is eliminated. However, we agree with the reviewer that not all per-study biases can be overcome. For example, DNA from a specific bacteria may not be extracted in one study and extracted in another. In the manuscript, the analysis focusing on IBD-specific and non-specific bacteria further attempt to overcomes this limitation by calculating the NRMD-based statistics only on studies where the ASV bacteria is present. Additionally, we use the direction of the effect (rather than the effect size) for the binomial test used to identify the non-specific bacteria, and therefore give equal weight to all studies, not dependent on the amplification level of the bacteria within each study. Doing this, we may lose some power (i.e. leading to false-negatives), combining the per-study NRMDs and treating them as independent measurements should not result in false positives. We have added a clarification about that in the results.

*3. Authors define a non-specific microbial alteration associated with multiple pathologies. Due to the use of 16S rRNA data, biological characterization is limited to the identification of taxa that are involved in these non-specific microbial alterations, while it lacks insights on metabolic pathways. Although this issue cannot be solved from this analysis, some discussions on the use of shotgun data should be added into the manuscript.*

**Response:** We agree with the reviewer and have this as a limitation in the discussion “Other limitations include the limited metadata characteristics across cohorts, and the use of 16S rRNA amplicon sequencing (rather than shotgun data) which limits the ability to identify taxonomy up to the strain levels and the bacteria associated metabolic pathways and functions”. As the reviewer suggested in point 7, we have referenced Gupta, V.K., et al., and expanded on the use of shotgun data for the generation of Gut Microbiome Health Index (GMHI).

*4. The identification of a universal dysbiosis index (UniDi) that can successfully differentiate between cases and controls (across multiple diseases) is interesting and give insights about a shared microbial response during the pathogenesis process. I feel authors should provide more examples/applications on the use of UniDi besides its application for faecal microbiota transplantation.*

**Response:** We thank the reviewer for his suggestion. As was previously stated (Gupta, V.K., et al.) such index can provide insight into one’s health status from a gut microbiome and for inferring the likelihood of disease independent of the clinical diagnosis. In Addition, this index can be calculated per samples and can be included in gut microbial personal report, which can also be followed longitudinally for variations linked with diet and environmental modification in clinical studies. We have added this to the discussion “Another application as already suggested for such index is for providing insight into one’s health status from a gut microbiome and for inferring the likelihood of disease independent of the clinical diagnosis. In Addition, this index can be calculated per samples and can be included in gut microbial personal report, which can also be followed longitudinally for variations linked with diet and environmental modification in clinical studies”.

*5. Effect sizes are calculated through NMRD. What if other strategies are employed for this? I suggest validating findings by considering effect sizes provided by other methods (such as LEfSe: Segata, Nicola, Jacques Izard, Levi Waldron, Dirk Gevers, Larisa Miropolsky, Wendy S. Garrett, and Curtis Huttenhower. "Metagenomic biomarker discovery and explanation." Genome biology 12, no. 6 (2011): 1-18)*

**Response:** We thank the reviewer for this suggestion. We have added LEFSE based LDA as another approach for the effect size calculation (Figure S2 and suppl dataset 1). Using this effect size (with a p-value cutoff of 0.1, as LEFSE does not include FDR correction) we identified 55 non-specific ASVs (41 lower and 14 higher in disease). Of the 41 ASVs lower in disease, 16 (39%) overlapped with our initial non-rarified NRMD reported ASVs lower in disease, and none overlapped with the initial non-rarified NRMD 31 ASVs higher in disease (p<0.0001). Similarly, of the 14 higher in disease, 6 (43%) overlapped with the non-rarified NRMD reported ASVs higher disease and none with 97 ASVs lower in disease (p<0.0001). As described in the response to comment #1 (rarefaction), we have also performed the direction overlap analysis for the LEFSE LDA effect size. Of the 128 bacteria identified as non-specific using the basic-NRMD analysis, 30/31 “higher in disease” and 95/97 “lower in disease” agreed when using the mean LEFSE LDA effect size. Advantages of using NRMD compared to LEFSE LDA are the fact that we can apply FDR correction within each cohort, and that NRMD uses ranked testing, which better handles outliers, thus leading to increased sensitivity of the basic-NRMD analysis.

*6. How does your findings relate to results presented in this paper (Duvallet, Claire, Sean M. Gibbons, Thomas Gurry, Rafael A. Irizarry, and Eric J. Alm. "Meta-analysis of gut microbiome studies identifies disease-specific and shared responses." Nature communications 8, no. 1 (2017): 1-10) that also tried to identify shared microbial responses?*

**Response:** As noted by reviwer1, our manuscript follows in the footsteps of Duvallet et al., Nature Communications 2017 and identifies general disease-associated bacteria and IBD-specific bacteria by using a metanalysis approach. This is also stated in the discussion “Another landmark metanalysis study took the second approach, started from the raw data of 28 published case-control studies, that sequenced different regions on the 16S gene (not limited to the V4 region), and also highlighted the depletion of health-associated bacteria across multiple diseases, and some specific microbial signal in colon cancer but not in IBD”. However, our analysis uses much more data than the original publications and an improved methodology (ASVs not OTUs at genus level, better control of country-study specific effects) and thus (hopefully) end up with more accurate set of disease-specific bacteria for UC and CD, as well as a large set of non-specific bacteria. In addition, we show that the non-specific bacteria should be considered when performing classification of disease vs. healthy samples, as in many cases different diseases are classified based on the same non-specific bacterial signal. Finally, we developed a generic dysbiosis index of general disease-associated bacteria (either lower or higher in multiple disease states) using machine learning and then show that it outperforms a CD-specific dysbiosis index. This was added more clearly to the discussion.

*7. Similarly to the previous point, the definition of the UniDi in the current work is quite similar to the definition of the Gut Microbiome Health Index (GMHI) performed in Gupta, Vinod K., Minsuk Kim, Utpal Bakshi, Kevin Y. Cunningham, John M. Davis, Konstantinos N. Lazaridis, Heidi Nelson, Nicholas Chia, and Jaeyun Sung. "A predictive index for health status using species-level gut microbiome profiling." Nature communications 11, no. 1 (2020): 1-16. Please comment on this.*

**Response:** We thank the reviewer for this comment. This work is referenced in the text (Gupta, V.K., et al.). This paragraph was added to the discussion as suggested “The concept of a dysbiosis index for estimation of the general host dysbiosis or healthy state was suggested in the landmark IBD study [2] and in recent shotgun sequencing dataset. The recent shotgun sequencing dataset used 4347 human stool metagenomes from 34 published studies across healthy and 12 different disease conditions highlighting 50 bacterial species more prevalent in pooling data from healthy vs. nonhealthy state cases. This index was generated and therefore can be applied on other shotgun datasets. We expand this idea using 12,838 subjects, 59 disease-cohorts, and 28 diseases and we generated an index that can be applied to dataset generated using 16S sequencing. Shotgun dataset can also inform regarding metabolic pathways and functions, which can be an interesting future approach for to define healthy vs. nonhealthy state pathways across diseases”.

**References**

Halsey, L.G., et al., *The fickle P value generates irreproducible results.* Nat Methods, 2015. **12**(3): p. 179-85.

Gupta, V.K., et al., *A predictive index for health status using species-level gut microbiome profiling.* Nat Commun, 2020. **11**(1): p. 4635.

**Second round of review**

**Reviewer 2**

I feel the authors have answered positively to my previous comments and modified the manuscript according to them.
